# Assessment of mutation probabilities of KRAS G12 missense mutants and their long-timescale dynamics by atomistic molecular simulations and Markov state modeling

**DOI:** 10.1371/journal.pcbi.1006458

**Published:** 2018-09-10

**Authors:** Tatu Pantsar, Sami Rissanen, Daniel Dauch, Tuomo Laitinen, Ilpo Vattulainen, Antti Poso

**Affiliations:** 1 School of Pharmacy, University of Eastern Finland, Kuopio, Finland; 2 Laboratory of Physics, Tampere University of Technology, Tampere, Finland; 3 Department of Internal Medicine VIII, University Hospital Tuebingen, Tuebingen, Germany; 4 Department of Physiology I, Institute of Physiology, Eberhard Karls University Tuebingen, Tuebingen, Germany; 5 Department of Physics, University of Helsinki, Helsinki, Finland; 6 MEMPHYS-Center for Biomembrane Physics, Helsinki, Finland; National Cancer Institute, United States of America and Tel Aviv University, Israel, UNITED STATES

## Abstract

A mutated KRAS protein is frequently observed in human cancers. Traditionally, the oncogenic properties of KRAS missense mutants at position 12 (G12X) have been considered as equal. Here, by assessing the probabilities of occurrence of all *KRAS* G12X mutations and KRAS dynamics we show that this assumption does not hold true. Instead, our findings revealed an outstanding mutational bias. We conducted a thorough mutational analysis of *KRAS* G12X mutations and assessed to what extent the observed mutation frequencies follow a random distribution. Unique tissue-specific frequencies are displayed with specific mutations, especially with G12R, which cannot be explained by random probabilities. To clarify the underlying causes for the nonrandom probabilities, we conducted extensive atomistic molecular dynamics simulations (170 μs) to study the differences of G12X mutations on a molecular level. The simulations revealed an allosteric hydrophobic signaling network in KRAS, and that protein dynamics is altered among the G12X mutants and as such differs from the wild-type and is mutation-specific. The shift in long-timescale conformational dynamics was confirmed with Markov state modeling. A G12X mutation was found to modify KRAS dynamics in an allosteric way, which is especially manifested in the switch regions that are responsible for the effector protein binding. The findings provide a basis to understand better the oncogenic properties of KRAS G12X mutants and the consequences of the observed nonrandom frequencies of specific G12X mutations.

## Author summary

The oncogene *KRAS* is frequently mutated in various cancers. When the amino acid glycine 12 is mutated, KRAS protein acquires oncogenic properties that result in tumor cell-growth and cancer progression. These mutations prevail especially in the pancreatic ductal adenocarcinoma, which is a cancer with an exceptionally dismal prognosis. To date, there is a limited understanding of the different mutations at the position 12, also regarding whether the different mutations would have different consequences. These discrepancies could have major implications for the future drug therapies targeting KRAS mutant harboring tumors. In this study, we made a critical assessment of the observed frequency of *KRAS* G12X mutations and the underlying causes for these frequencies. We also assessed KRAS G12X mutant discrepancies on an atomistic level by utilizing state-of-the-art molecular dynamics simulations. We found that the dynamics of the mutants does not only differ from the wild-type protein, but there is also a profound difference among the different mutants. These results emphasize that the different *KRAS* G12X mutations are not equal, and thereby they suggest that the future research related to mutant KRAS biology should account for these observations.

## Introduction

The small GTPase protein KRAS is a signal-transducing protein, which binds GDP in its inactive state and GTP in its active state [1]. The gene *KRAS* is frequently mutated in various human cancers. The mutation is most often, in about 86% of the cases [2], found at G12. In fact, every missense mutation at G12 (G12X) is oncogenic. The oncogenic properties associated with *KRAS* G12X mutation are characterized by the deficiency of the intrinsic GTPase activity and the insensitivity for GTPase-activating proteins (GAPs) [3,4]. These alterations lead to increased KRAS signaling, as there is more active GTP-bound protein present. Still, the mutant KRAS undergoes GDP–GTP cycling [5]. The basis of the specific G12X mutation frequencies has remained unclear, except for the G12C transversion mutation (c.34G>T) associated with smoking in lung cancer [6,7].

An interesting discrepancy among KRAS G12X mutants is observed in their intrinsic GTPase activity [8]. The G12A mutation exhibits the most hindered intrinsic hydrolysis (~1% compared to the wild-type), whereas the G12C mutation displays the least hindered activity (~72%). All G12X mutants, however, show insensitivity to GAPs that accelerate hydrolysis [8]. Importantly, not only RAS G12X mutants exhibit a discrepancy in GTP hydrolysis, but they also give rise to differences in the preferred signaling pathway (in terms of effector protein binding) [9,10]. This behavior was first observed in NSCLC cell lines [9], where KRAS G12D showed activation of PI3K and MEK signaling, while G12C and G12V mutants exhibited activated RalGDS-pathway and diminished growth factor-dependent Akt activation. Furthermore, an NMR study revealed different binding preferences for mutant HRAS G12V compared to wild-type HRAS, with various effector proteins [10]. Here, HRAS G12V showed reduced interactions with Raf and enhanced binding with RalGDS. However, given that the non-hydrolysable GTP-analog GNP was used in the study, the difference is not due to impaired hydrolysis. Similarly with HRAS, KRAS G12X mutants exhibit reduced affinity to Raf compared to wild-type [8]. The G12D, G12R, and G12V mutants display highly reduced affinity to Raf, while the affinity of G12A is only moderately reduced. Interestingly, the affinity of the G12C mutant is similar to that of wild-type.

To bind RAS, the effector proteins use a ubiquitin (UB)-like fold: a RAS-binding domain (RBD) or a RAS-association domain (RA) [11,12]. While KRAS has not been co-crystallized with any of its effector proteins, distinct effector proteins have been resolved in complex with HRAS: RalGDS (PDB ID: 4G0N) [13], Raf-1 (PDB ID: 1LFD) [14], PI3Kγ (PDB ID: 1HE8) [15], PLCε (PDB ID: 2CL5) [16], RASSF5 (PDB ID: 3DDC) [17] and AF-6 (PDB ID: 6AMB) [18]. These effector proteins bind to HRAS on top of its switch regions: switch-I (residues 30–40) and switch-II (residues 58–72), and the binding conformation of HRAS is almost identical in all of the complexes (S1A Fig). Given this, and since the G12X mutation is far from the binding interface (S1B Fig), Smith and Ikura [10] proposed that the discrepancies in the effector protein binding profiles of the mutants are due to altered switch dynamics. Overall, switch-I displays highly dynamic characteristics manifested as two different states when GTP is bound to RAS, and the distribution between these states is altered in mutants [19–22]. Given that the switch regions in HRAS and KRAS are identical (S1C Fig), their expected binding mode to their effectors is alike. A model of KRAS in complex with A-Raf-RBD tethered to a lipid-bilayer nanodisc suggested by NMR data agrees with this binding mode [23]. At the cellular level, the isoform specificity to effector proteins is primarily determined via membrane interactions [24], but the differences among RAS isoforms’ absolute effector protein binding affinities rise from allosteric effects [25]. It was observed that even a single point mutation in RAS (Q61L) has long-range effects on dynamics and alters effector protein interactions [13].

Previous molecular dynamics (MD) simulation studies of KRAS at microsecond timescales have mainly focused on the dynamical differences between the three wild-type RAS isoforms (HRAS, KRAS, NRAS) [26], differences among selected KRAS and HRAS mutants [27,28], the role of the hypervariable region (HVR) [29], KRAS’s membrane association or orientation [30–32], and KRAS oligomerization on the membrane [33]. The total simulation times of these studies were in the range of 1–8 μs, which is reasonable but likely not sufficient to unravel long-time dynamics associated with slow conformational changes. More importantly, there is a lack of comprehensive atomistic MD simulations of all KRAS G12X mutants with extensive simulation times, allowing a reliable analysis for the differences in structure and dynamical behavior between the wild-type and the mutants, especially in the effector protein binding interface.

What is the underlying cause for the broad range of different G12X mutations? How do these distinctly different mutations manifest themselves in the structure, dynamics, and function of KRAS? This knowledge is crucial to understand KRAS oncogenesis and to develop future therapies targeting mutant KRAS harboring tumors. Therefore, in the present study we first assessed to what extent G12X mutation frequencies are explained by mutation probability. Intriguingly, an outstanding mutational bias emerged from the data. We next employed state-of-the-art atomistic MD simulations (total simulation time 170 μs) to study the dynamical behavior of KRAS with its natural ligands (GDP, GTP) bound, both in the wild-type KRAS and with all existing oncogenic G12X mutations. The results provided compelling evidence that mutations alter the dynamics of KRAS, that the alteration is mutation specific, displays allosteric characteristics, and that the alteration is manifested especially in the effector protein-binding interface. Furthermore, our data suggest that the observed mutational bias and the oncogenic properties of the individual KRAS G12X mutants are caused, at least in part, by mutation-specific altered dynamics.

## Results

### Distribution of *KRAS* G12X mutations is not random

First, to perceive up-to-date data of *KRAS* G12X missense mutation frequencies, we compiled data from the Catalogue of Somatic Mutations in Cancer [2]. A total of 32,654 tumor samples identified with a *KRAS* G12X missense mutation were found from the database. For our analysis, we included only tissues that exhibited these mutations >10%. This status is displayed in eight tissue types, which in total comprised 31,251 positive samples (95% of all *KRAS* G12X mutations in the database). The large intestine (18,174), the lung (5,640), and the pancreas (5,528) were observed to have numerous positive samples, whereas the biliary tract, the endometrium, the ovary, the peritoneum, and the small intestine comprised altogether only 2,085 positive samples.

**Fig 1:**
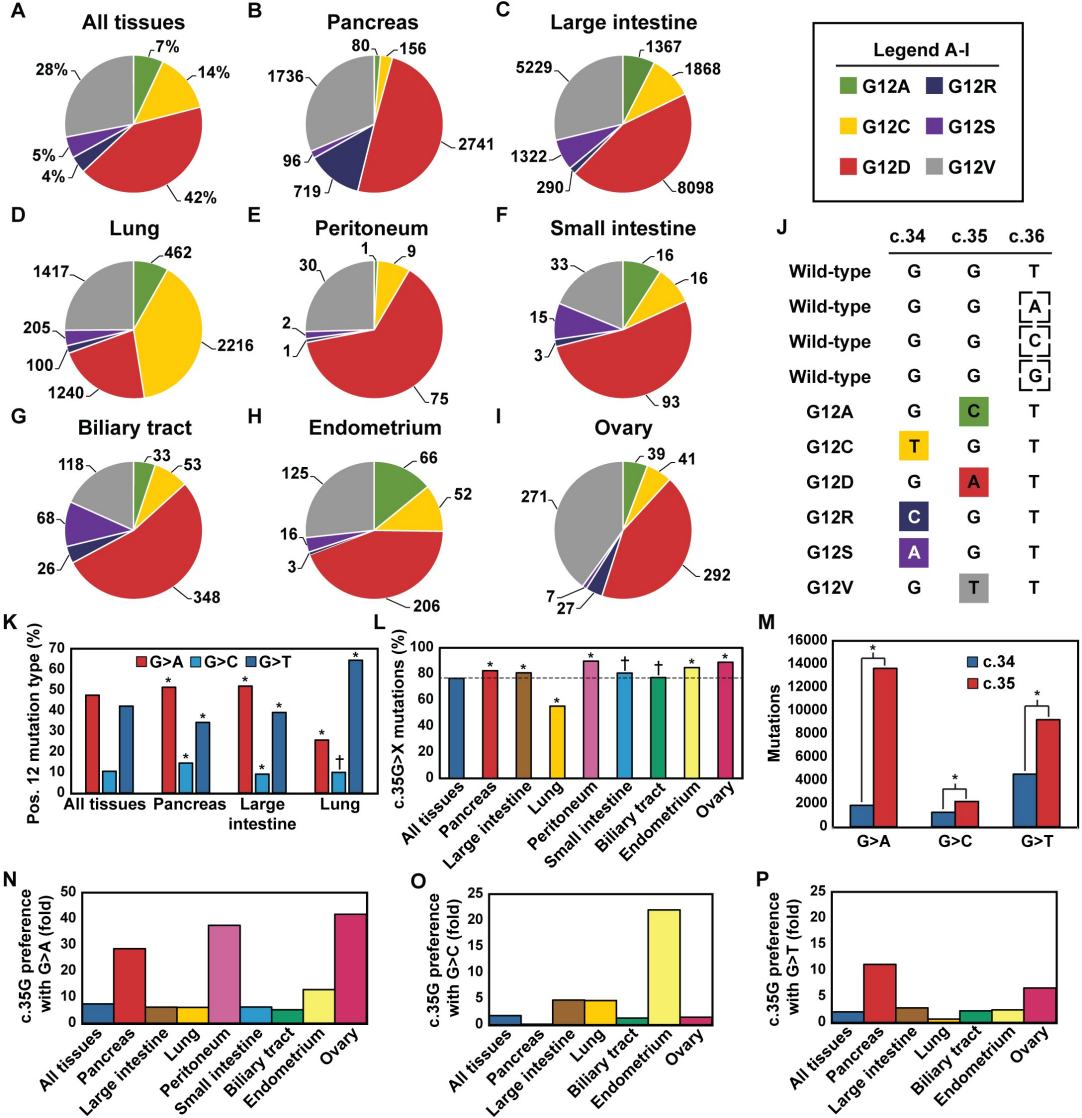
The occurrences of specific *KRAS* G12X mutations vary among different tissues, and tissues exhibit individual preference in mutation type and position. The occurrence of specific mutations in **(A)** all tissues, (**B**) the pancreas, (**C**) the large intestine, (**D**) the lung, (**E**) the peritoneum, (**F**) the small intestine, (**G**) the biliary tract, (**H**) the endometrium, and (**I**) the ovary. Numbers shown in panels **B-I** indicate the numbers of observed positive tumor samples. In panels **B-I**, the data are ordered from the highest occurrence (%) of a G12X mutation (panel **B**) to the lowest (panel **I**). Data have been collected from the COSMIC database [2] v.79 (http://cancer.sanger.ac.uk/cosmic/). (**J**) *KRAS* G12X single point mutations occur if c.34 or c.35 is mutated. (**K**) Mutation types observed in all tissues, the pancreas, the large intestine, and the lung. (**L**) Fraction of position c.35G mutations compared to all the mutations averaged over all tissues, and found in individual tissues. (**M**) Positional mutation preference of specific mutation types in c.34 and c.35. (**N-P**) Position mutation preference characterized through c.35G over c.34G in specific tissues with (**N**) G>A, (**O**) G>C, and (**P**) G>T mutations. Tissues with less than 50 positive samples (the peritoneum, the small intestine) have been omitted from the panels **O** and **P**. Statistically significant differences in the panels **K**-**M** compared to other tissues or position are indicated with an asterisk, * *P<0*.*001*; † = non-significant (Fischer exact test).

A point mutation in *KRAS* G12X may result in one of six possible missense mutations (Fig 1J). However, instead of being evenly distributed, these specific mutations display considerable variation (Fig 1A). Overall, G12D (42%), G12V (28%), and G12C (14%) mutations are very common, whereas G12A, G12R, and G12S are less popular. When the relative fractions of these mutations are considered in different tissues, they are readily observed to vary significantly (Fig 1B–1I) [2]. For instance, the G12R mutation is observed in the pancreas with a probability of 13%, while in the small intestine it appears in less than 2% of the cases. The predominating mutations are G12D and G12V, the lung being an exception with G12C standing as the most abundant mutation.

In a G12X missense mutation, the guanine (G) base in c.34G or c.35G is substituted to adenine (A), cytosine (C), or thymine (T) (Fig 1J). This base-substitution type exhibits variation (Fig 1K). G>A and G>T mutations (47.4% and 42.1%, respectively) occur very often, while the G>C mutation (10.5%) takes place more seldom. These occurrences display some variation in different tissues. Particularly the lung differs from other tissues with a higher G>T fraction and a diminished proportion of G>A mutations (*P* < 0.001). Meanwhile, in the pancreas the probability of the G>C mutation is increased (*P* < 0.001). Moreover, as all of the G12X mutations occur in the first or the second guanine of the codon (c.34G, c.35G) (Fig 1J), we ascertained if there is a mutational bias between these positions. Interestingly, 76.6% of the G12X mutations are c.35G>X mutations (G12A, G12D, G12V) and only 23.4% are c.34G>X mutations (G12C, G12R, G12S) (Fig 1L). In fact, all tissues, except for the lung (55.3%), display 77–90% of c.35G>X mutations. The positional mutation preference for c.35G>X seems to be the highest with a G>A mutation (>7x), whereas G>C or G>T exhibit nearly twofold preference, 1.75- and 2.03-fold, respectively (Fig 1M). A few exceptions in the c.35G>X preference, however, appear in specific tissues. In the pancreas, the G>C mutation occurs nine times more often in c.34 than in c.35 (Fig 1O). As for the G>T mutations, the lung is the only tissue where c.34 is preferred (>1.5x) (Fig 1P). All tissues, interestingly, exhibit over fivefold c.35 preference in G>A mutations (Fig 1N). Above all, the pancreas (>28x), the peritoneum (>37x) and the ovary (>41x) exhibit the most prominent preference for the G>A mutation in c.35.

We evaluated how random the occurrences of the specific G12X mutations are. To this end, we used the transition:transversion mutation ratio as a figure of merit, and compared this figure to a value of 2.3, which is the ratio for missense mutations observed in large-scale genomic analyses [34,35]. If the mutations would take place randomly, G12D and G12S mutations should be the most abundant mutations as they are transition mutations (S2 Fig). G12D mutation is consistent with this view, as it occurs very often in all tissues. Meanwhile, G12S is not consistent with this behavior at all, as it occurs in tumors, perhaps surprisingly, very rarely. Also, regardless of the tissue type, the G12V mutation is overexpressed compared to values expected based on the assumption of random occurrences. Concluding, the mutations’ probabilities of occurrences are not consistent with a transition:transversion mutation ratio based on a random process.

Since local DNA-sequences have clearly a major influence on the mutation probability, a sequence-dependent basis for the observed non-random mutations may exist. The T*GGT* sequence lacks a typical hotspot mutation region, such as a CpG site [36]. However, an adjacent GG region is a susceptible site for a mutation [37,38]. The oxidation of guanine by endogenous reactive oxygen species may also result in DNA mutation [39]. Both guanines, the 5’G and the 3’G in a GGT-sequence, are found to act as sites for frequent one-electron oxidation reactions, and they exhibit only a minor difference (0.05 eV) in their vertical ionization potential [37]. The oxidation can further transform guanine to 7,8-dihydro-8-oxoguanine (8oxoG), which promotes especially the G>T transversion mutation [40], and the G>T mutations take place on a regular basis (Fig 1K). Interestingly, studies of DNA-adduct formation by exogenous agents have resulted in adduct formation only in c.34G, and not in c.35G [41,42]. Finally, cigarette smoking promotes G12C mutations exhibited regularly in the lung tissue (Fig 1D and 1K) [7]. Concluding, there are several potential mechanisms able to alter the mutation profile of guanine, thereby leading to the data we discussed above.

### Atomistic molecular dynamics simulations reveal that the switch regions in KRAS are highly mobile

To understand how G12X mutations affect KRAS functionality, we conducted a total of 170 μs (85 x 2 μs) atomistic MD simulations of wild-type KRAS and all G12X missense mutants, with GDP and GTP. Each individual system was replicated five to ten times starting from different initial conditions (S1 Table).

In the simulations, we observed no differences in the dynamics of the residue 12 (or in its vicinity), which appeared to be extremely stable. In contrast, the switch regions (switch-I and switch-II) exhibited highly dynamic behavior demonstrated by the root-mean-square fluctuation (RMSF) analysis, which revealed major fluctuations in the protein in these regions (S3 and S4 Figs). Nevertheless, there were no evident differences between the different systems, as generally the individual replicas displayed variation as much as the different systems. Only with GDP, the G12A and the G12S display a different RMSF profile in the switch-I region.

### The bound ligands GDP and GTP affect KRAS dynamics

**Fig 2:**
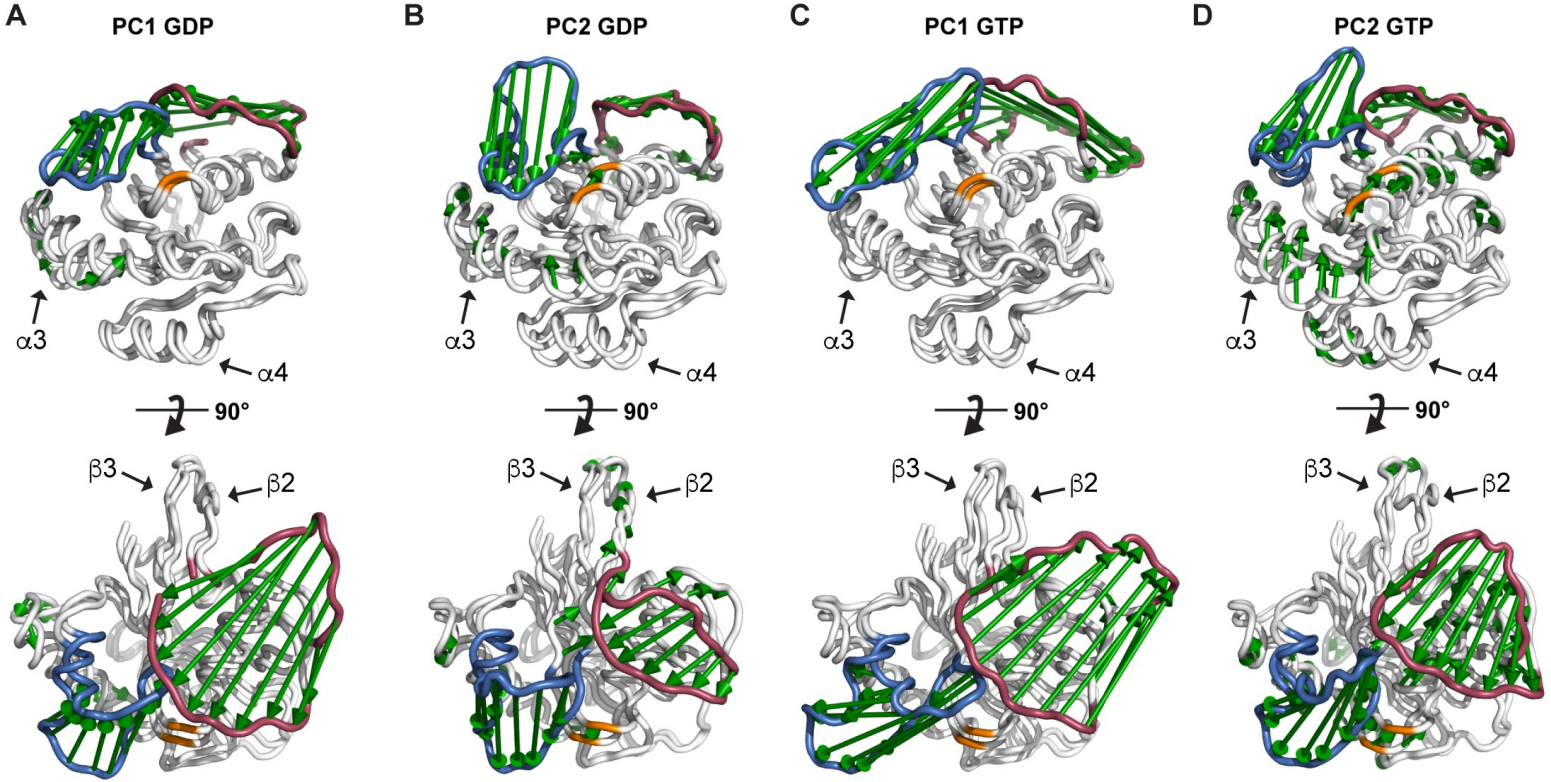
GDP- and GTP-bound systems exhibit distinctly different dynamics. The extreme movements of the principal components PC1 and PC2 in all (**A, B**) GDP systems and (**C, D**) GTP systems. Color coding: residue 12 (orange), switch-I (residues 30–40, red), and switch-II (residues 58–72, blue).

To gain better insight into the protein dynamics, we conducted principal component analysis (PCA) [43] with an objective to find the most significant large-scale motions of KRAS. PCA revealed that the greatest dynamic movements in the protein occur in the switch regions (Fig 2, S5A Fig). Furthermore, the most significant principal components 1–3 (PC1-3, see S5B Fig for contributions) highlight that there are strong differences between GDP-bound and GTP-bound systems. PC1 of the GTP-bound systems displayed movement only in the switch regions, whereas PC1 of the GDP-bound systems exhibited additional movement also in the α3-helix. PC2 of GDP-bound KRAS revealed the movement in the switch regions and also extensive motion in the α3-helix, the hairpin loop between the β2- and β3-sheets, and the P-loop (see S1D Fig). PC2 of GTP-bound KRAS in turn brought out the movements observed in GDP-bound systems, and further also the motion in the α1- and α4-helices, and in the turn near the SAK-motif. These observations indicate that the key to resolve the changes in protein dynamics is the γ-phosphate.

Notably, the α4-helix motion is only observed with GTP bound systems (PC2). This observation is in agreement with the experimental results by Mazhab-Jafari et al. [23]. They observed that the GDP-bound KRAS drives the protein in the “exposed” configuration on the membrane, where the α4-helix is located in close proximity to the membrane (PDB ID: 2MSC). This would indicate that the dynamical stability of the helix is important for this state.

### Individual mutants display unique dynamics

**Fig 3:**
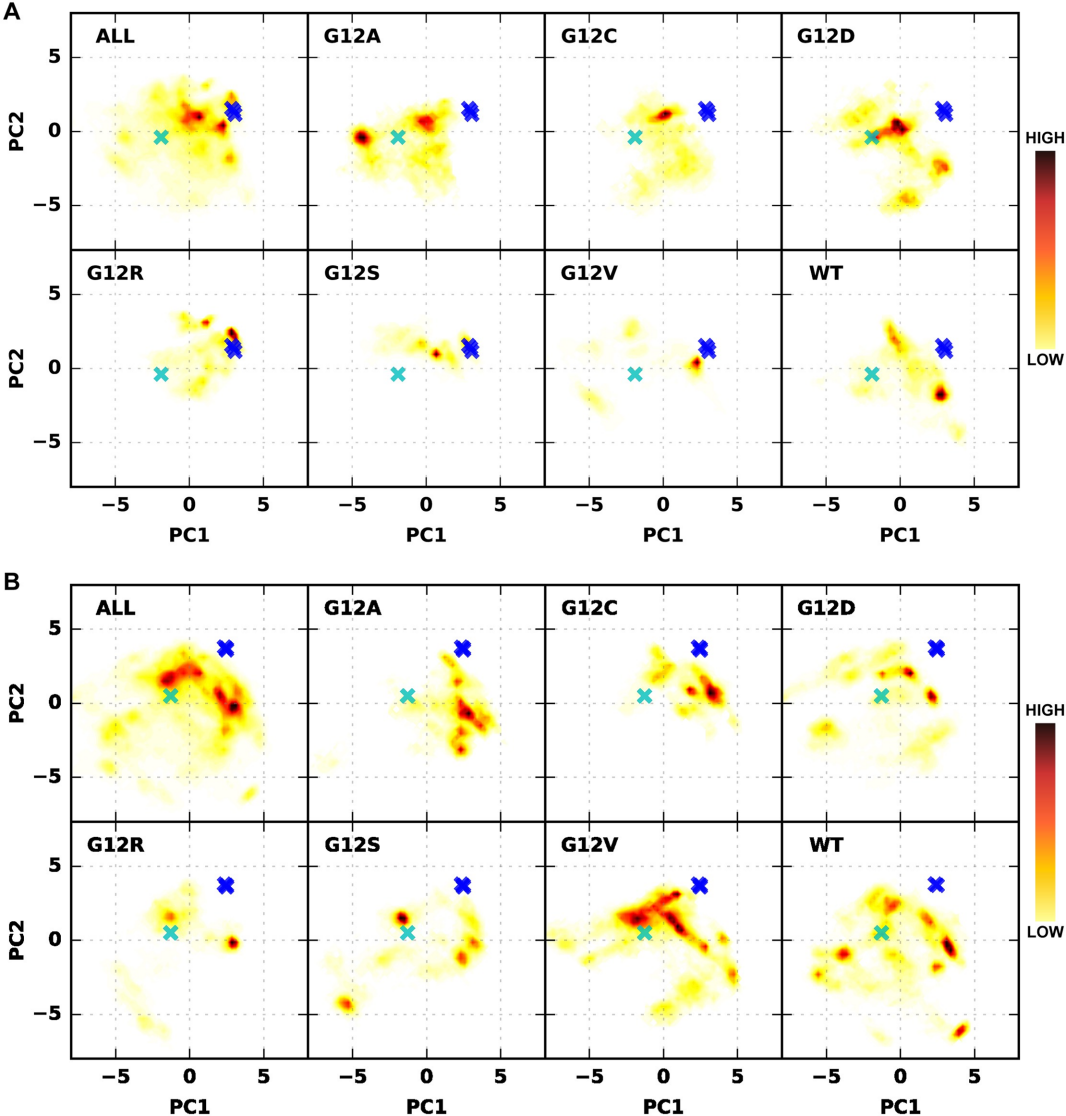
Dynamics of the mutants characterized by their principal components display individual profiles. PCA (backbone) score plots (heat map) of (**A**) GDP-bound and (**B**) and GTP-bound systems. Top-left boxes comprise all the systems with (**A**) GDP or (**B**) GTP. For conformational reference, the backbone conformation of RAS from the RAS–effector and RAS–GEF complexes is included in the plots, where switch-I and switch-II are in a totally closed conformation (blue crosses; from RAS–effector protein complexes) or switch-I is in a fully open conformation (cyan crosses; from a RAS–GEF complex). Reference RAS structures were obtained from HRAS–effector protein complexes (PDB IDs: 1HE8, 1LFD, 4G0N) and from the HRAS–Sos complex (PDB ID: 4NYJ).

In order to ascertain dissimilarities between the different systems, we next generated score plots for the principal components PC1-3 (Fig 3, S5C and S5D Fig). The results highlight dissimilarities between the wild-type KRAS and the mutants, as well as between GDP- and GTP-bound proteins. Interestingly, in all of these systems, only the G12R and G12S mutants with GDP appear to reside in the closed switch-I conformation, whereas all other systems eluded this conformation. Even more interestingly, both of these mutants evaded this conformation when they were bound to GTP. The fully open conformation of switch-I appears to be more accessible to the systems, especially with the G12D mutant with GDP. Moreover, wild-type in both GDP- and GTP-bound systems seems to have a unique state with a high-scoring value (+3 and +4) in PC1 and a low-scoring value (-2 and -0.5) in PC2. Taken together, the results show for all the mutants that the profile of their large-scale motions differs from wild-type regardless of the bound ligand, and that the profile is also unique to each mutant.

We extended the analysis by carrying out PCA for each system to illustrate the differences in their dynamics (S6–S9 Figs). The individual PCA analyses highlight not just variation in the switch region movements among the systems, but they also show that specific systems display more dynamical behavior in the α3-helix, hairpin loop between the β2- and β3-sheets, the α4-helix, the loop between β5-sheet and α4-helix, and in the SAK-motif (residues 145–147) regions. For example, in GTP-bound systems only the G12A, G12D, G12R, and G12V mutants exhibited movement in the α4-helix in their PC1 or PC2. Interestingly, these are also the systems that exhibit clearly diminished Raf affinity [8]. Also, the G12R mutant with GTP displayed notably reduced movement in the switch-II region in both PC1 and PC2.

### Hydrophobic hubs form an allosteric interaction network throughout KRAS

**Fig 4:**
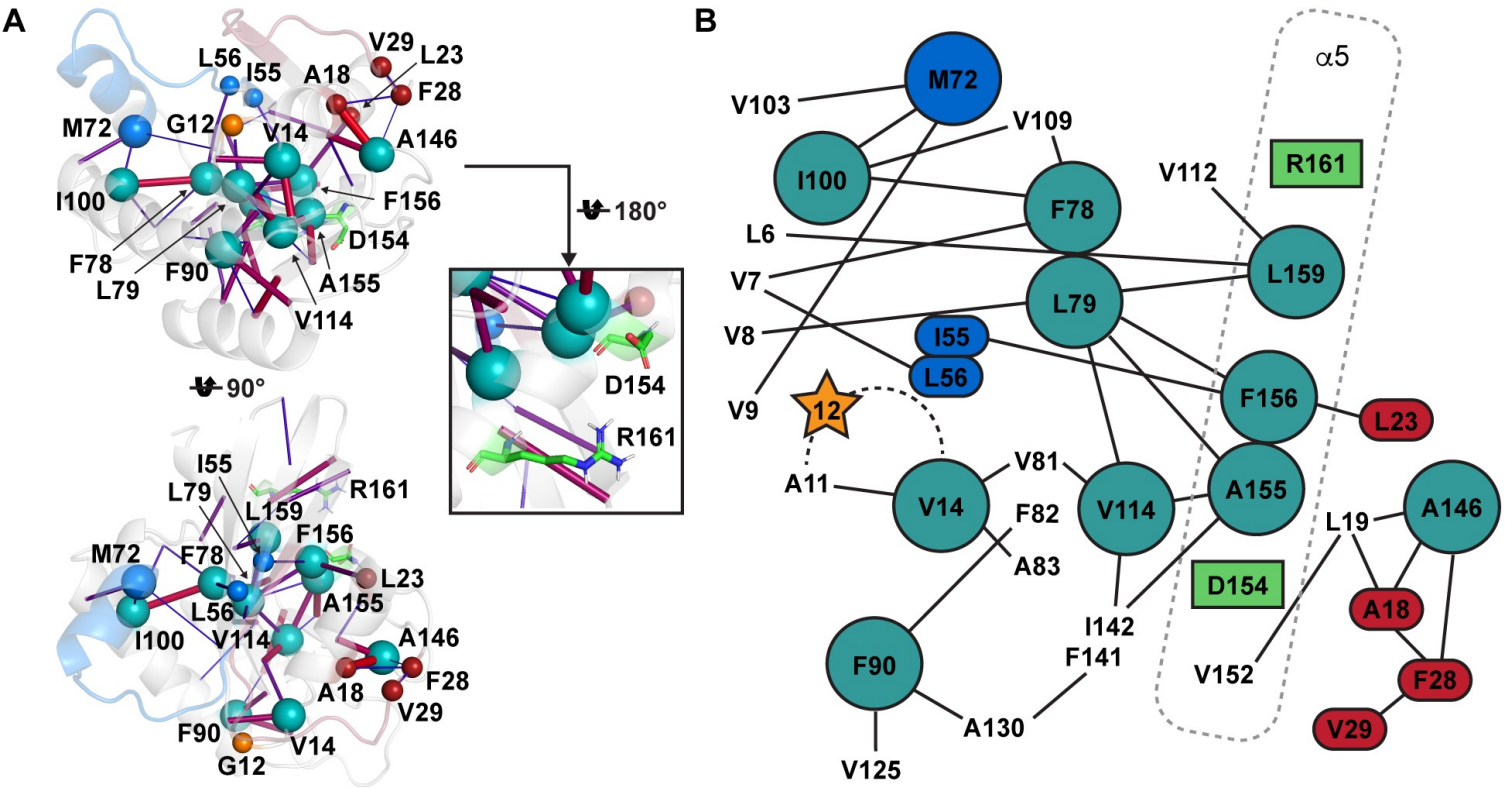
The hydrophobic interaction network of KRAS. The interaction network is represented for the GTP-bound wild-type KRAS (**A**), where hydrophobic hubs are displayed as large teal spheres (except for M72 depicted as blue). The hubs are defined by the criteria that each hub displays at least three hydrophobic contacts (>10%). The hydrophobic interactions (>10%) are depicted with cylinders, where the frequency of interaction is depicted in a scale from blue and thin to red and thick for low and high frequencies, respectively. The hydrophobic residues connected to the network that can influence the switch-I and switch-II dynamics are shown in small red and blue spheres, respectively. Moreover, the hub M72 is directly part of switch-II (large blue sphere). The salt-bridge forming residues D154 and R161 are displayed as green sticks. In the 2D representation of the hydrophobic hub network (**B**), the hydrophobic interactions (>10%) are shown with lines, and in addition to the hubs (spheres) all the hydrophobic interacting residues to the hub network are shown (in GTP-bound wild-type). The P-loop is indicated with a dashed line.

Even though there are no additional direct interactions from the mutated side-chains of G12X, a mutation in this position inflicts a change to the dynamics in the distant sites of KRAS that were highlighted by the PCA analysis. To investigate this, and to identify possible interaction network routes in KRAS, we conducted an interaction network analysis [44]. Interestingly, we identified a hydrophobic hub network in KRAS that indeed connects the distant sites in the structure and is able to convey these effects in an allosteric way (Fig 4). Therefore, a change in KRAS dynamics in one of the hydrophobic hubs could traverse through this network even to the distant sites. This hub network is comprised of 11 hubs: V14, M72, F78, L79, F90, I100, V114, A146, A155, F156 and L159. One of these hubs, V14, is located in the P-loop, in the close proximity of G12X.

This hub interaction network is highly distorted in G12A and G12S mutants (S10 and S11 Figs). The distortion in these mutants is further not ligand dependent. For example, in the V14 hub, the G12A and G12S mutants lack the interaction to A81 (<2% vs. wild-type 26.9% and 39.7%, with GDP and GTP, respectively), and also display highly diminished interaction to A11 compared to the wild-type KRAS (S10A Fig). From the V114 hub, these mutants lack interactions to A155 and L79, but instead have a strengthened interaction to I142 and a totally new interaction to L113, which is not displayed by other systems (S11A Fig). From the hub A146, they lack the interactions to A18 and L19 (S11B Fig). From the hub A155, both lack the interactions to V114, L79, and I142, but instead they have an elevated interaction to F156, and the G12A has an additional interaction to V152 (S11C Fig).

In contrast to G12A and G12S, the other mutants (G12C, G12D, G12R, and G12V) seem to follow more closely the wild-type’s interaction patterns. However, selected interactions are shifted even with these mutants, although not that extensively as observed with G12A and G12S. For instance, in the hub M72 located in the switch-II region the interaction patterns are shifted with G12C, G12R, and G12V (S10B Fig). Interestingly, the GTP-bound G12D mutant displays almost identical interactions with the wild-type.

We also noticed that the frequency of the salt-bridge between the residues D154–R161 was altered in different systems (S11F Fig). Both of these residues are located in the α5-helix, in the close proximity of three hydrophobic hubs: A155, F156 and L159 (Fig 4). With the wild-type KRAS this salt-bridge is more stable with GDP (69.1%) than with GTP (46.4%). Meanwhile, again with the G12A and G12S mutants this salt-bridge is highly distorted (4.5%–20.6%), regardless of the bound ligand.

### Markov state models confirm altered dynamics in selected mutants and reveal specific metastable state distributions

As discussed above, the PCA and the interaction network analysis suggest that the protein dynamics is altered among the systems, yet in some obscure manner. To gain better insight into the origin of these differences in wild-type and mutant GTP-bound systems (active KRAS), we analyzed the simulation data by constructing Markov state models (MSMs) [45,46] to explore the long-time statistical conformational dynamics of KRAS. The goal here was to identify the clusters of highly identical protein conformational states, here called metastable states, and to explore how the conformations of the wild-type and the mutant proteins are distributed between these metastable states. For the analysis, we selected the wild-type KRAS together with the most abundant mutants G12D and G12V, and the intriguing G12R mutant, which displays a highly variable distribution in the different tissues (Fig 1).

**Fig 5:**
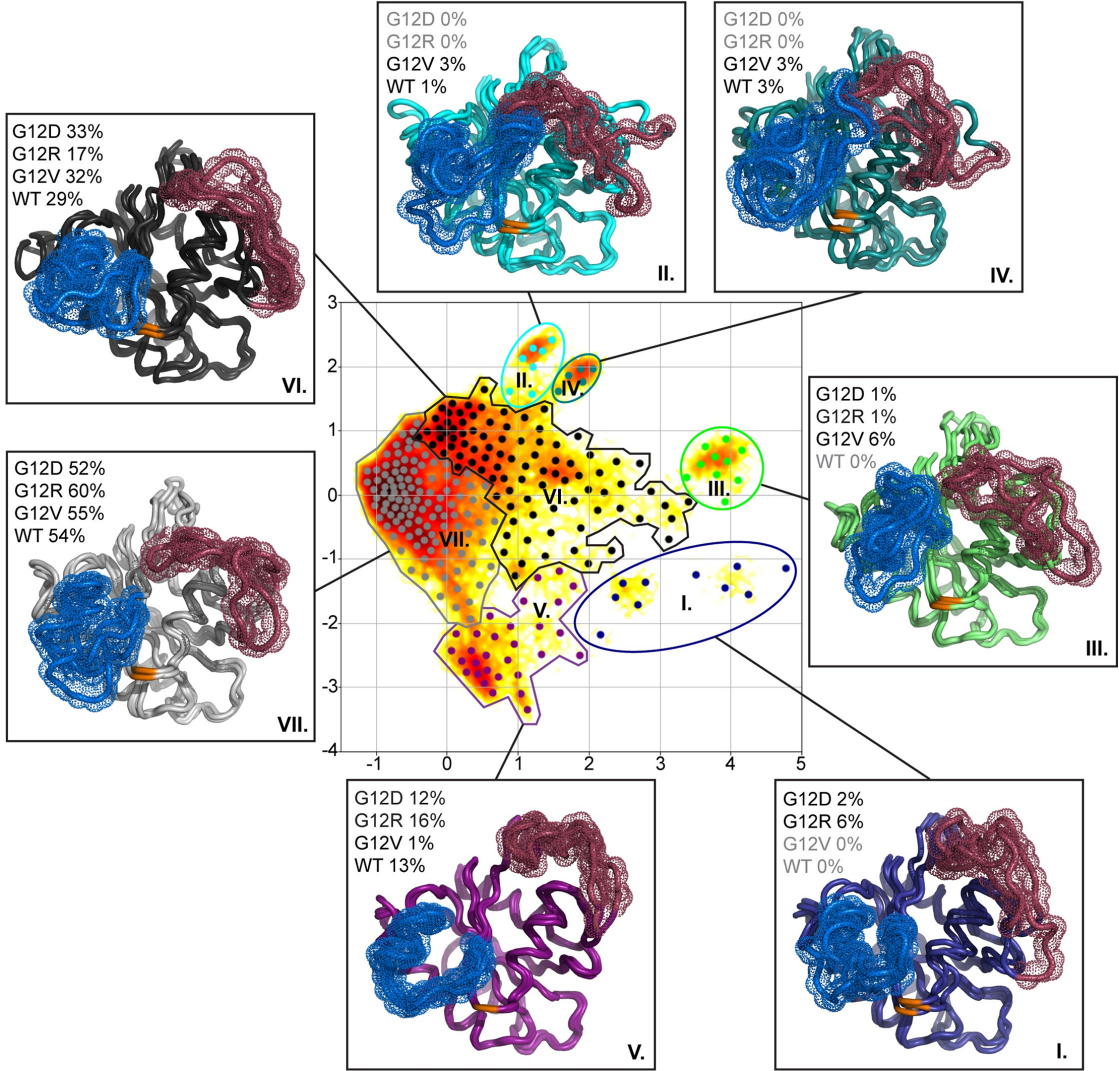
The seven metastable states identified by the Markov state model. In the middle is the time-lagged independent component analysis (TICA) plot showing seven clusters, each of which corresponds to one metastable state (I–VII). The metastable states are classified by borderlines, and the “microstates” (dots) in each metastable state are colored with the same color. The seven boxes around the middle describe the seven metastable states found in the MSM analysis, each box illustrating three representative protein conformations (generated using MSM), which identify the residue 12 (orange), and also the switch-I (red) and switch-II (blue) regions depicted by their backbone’s molecular surface (dots). In each metastable state, the occupation percentage by wild-type and individual mutants is displayed next to the conformations. For each case (wild-type, G12D, G12V, G12R), normalization of the percentages is done such that the sum over the seven metastable states adds up to 100%.

The MSM analysis identified seven metastable states represented schematically in Fig 5. Overall, all systems populate frequently two of the states: the states VI and VII (77–87%). The less populated metastable states I-IV are specifically represented among the systems. The metastable state I is quite unique for G12R (6%) and the state III for G12V (6%), whereas the other systems are mostly absent from these two states. The metastable state IV is only present in wild-type (3%) and in the G12V mutant (3%). The moderately populated metastable state V, where switch-II appears in a fixed conformation, is rarely observed with the G12V mutant (1%), whereas it is similarly represented among the other mutants and the wild-type (12–16%). In fact, the switch-II conformation appears to be closed in the effector protein complexes (S1A Fig). However, none of the observed metastable states corresponds to this specific switch-II binding end-point conformation.

The states can be further divided in four groups based on their switch conformations (Table 1). The states I and V as combined form the first group, where switch-I appears to be in a fully open conformation and switch-II in a fixed conformation. This group is frequently occupied by G12R (24%), whereas it is mostly absent from G12V (1%). The states VII and VI form individually the second and the third groups, in respective order. In the state VII, switch-II is in a fixed conformation and switch-I is more closed compared to the first group. This group is more frequently populated by G12R. In the state VI, switch-I is open and switch-II is in a mixed conformation between the fixed and perpendicular conformations. This state is clearly less populated by G12R compared to the other systems. The fourth group, where the switches appear in a perpendicular conformation, is frequent with G12V (12%), whereas the other mutants rarely visit this state (1%).

**Table 1:** Distribution of conformational groups.

Group	Switch-I / Switch-II conformation	States	G12D	G12R	G12V	WT
1	Fully open / fixed	I, V	14%	24%	1%	13%
2	Semi-open / fixed	VII	52%	60%	55%	54%
3	Open / mixed	VI	33%	17%	32%	29%
4	Perpendicular	II, III, IV	1%	1%	12%	4%

Of all the mutants, G12D displays the most similar metastable state population distribution compared to wild-type (Fig 5, Table 1). This is most evident in the most populated states (states IV, VI, and VII), yet G12D also differs from wild-type in the less populated states (I–IV). In contrast, the conformations of G12R are clearly shifted towards the fixed switch-II states, whereas G12V is shifted away from these states towards the perpendicular states. The results suggest that for G12V the shift among the states is due to the mutant’s lipophilic character, which may cause changes in solvent organization preventing specific switch-II conformations. Finally, it is exceptional that while wild-type does not populate the metastable states I and III at all, there are mutants (G12R, G12V) whose population in these states is significant (about 6%). This summarizes the main message: the conformation distribution of KRAS mutants includes conformations not occupied by wild-type, and these conformations are also mutation specific.

## Discussion

Although frequently observed in cancer, not only is the basis for the specific frequencies of *KRAS* G12X mutations poorly understood [47], but also the effects of these specific mutations on a molecular scale are not clear. To the best of our knowledge, this is the first study to assess *KRAS* G12X mutation probabilities, and to understand how they are associated with the observed mutation frequencies.

Generally, the mutation frequencies have an explanatory basis. For instance, chemical characteristics of c.35G explain the enrichment of the G12V mutations by oxidation. However, complex mutation distributions are displayed by the tissues, and we conclude that some of the observed frequencies cannot be explained simply by the mutation probability. For example, there is no clear explanation why, on average, a mutation occurs five times more probably in, e.g., c.35G than in c.34G. One plausible explanation is that the 3D-environment in the DNA-sequence may aid the c.35G mutations to evade DNA-repair mechanisms. In fact, the structures of DNA in complex with N-glycosylase/DNA lyase (*OGG1*), which is a base-excision repair enzyme for 8oxoG, exist in a catalytically active form for 8oxoG that is adjacent to guanine only in the 5’-position in -AGGT- sequences (S12 Fig) [40,48–51]. Correspondingly, this 5’G position in the *KRAS* sequence (-TGGT-) represents the c.34G position, thus suggesting that the c.34G position is more susceptible for DNA-repair. Nevertheless, this observation holds true only for the G>T transversion mutation. For the other mutations and their repair mechanisms, the positional bias needs to be investigated, especially for the G>A transition mutation, which holds the strongest bias in favor of c.35G mutations (>5x in all tissues). Furthermore, exceptions or an enhanced preference in c.35G for specific mutations in particular tissues were observed. For instance, it seems that either the advantage for G12D or the disadvantage for G12S, or both, exists in the pancreas, given that there is a 28-fold preference for G>A mutations for c.35G over c.34G. Similarly, the G12R mutation displays an advantage in the pancreas, while G12A is perhaps disadvantageous, given that there is a 9-fold preference for c.34G over c.35G in the mutation probability in the G>C mutations. Altogether, these data suggest that specific mutations are advantageous or disadvantageous depending on the cellular and tissue environments. Therefore, we hypothesize that the biochemical and biophysical differences among mutants, resulting in signal-transducing differences, may explain, at least in part, the observed mutational bias.

To gain insight into these observed discrepancies among the mutants on a molecular level, we carried out a comprehensive all-atom MD simulation study of all KRAS G12X missense mutants. We found that mutations have a profound effect on the dynamics of KRAS. In particular, we observed that the switches are highly dynamic. This conformational flexibility revealed through atomistic simulations is consistent with ^31^P-NMR spectroscopy studies of RAS proteins [21,52], while the published KRAS crystal structures do not unlock this behavior. Even in our extensive analysis of the long-timescale simulation data the differences in the dynamics were not readily visible. This is not surprising given that even though the binding affinity of a specific mutant toward an effector protein is increased or diminished, the ability to bind still exists [8]. This suggests that the changes in protein dynamics are quite subtle and difficult to quantify. In our work, we unlocked this issue through the analysis of the simulation data using PCA, interaction network analysis and MSMs that indeed revealed the differences, not only between the wild-type and the mutants, but also between the mutants.

In order to capture the subtle differences among the mutants, we kept our MD simulation systems realistic but sufficiently simple, enabling the extended simulation times close to 200 μs in total. Even though the HVR and the cell membrane were absent from our simulations, we recognize that these elements have a substantial influence on KRAS dynamics [53,54] and signaling [55]. We therefore cannot deduce whether some of the observed mutational effects attenuate or amplify through these factors. However, effects related to the cell membrane remain to be explored in follow-up studies.

Importantly, we identified the hydrophobic hub interaction network that is able to convey the shifts in KRAS dynamics throughout the whole structure in an allosteric manner. The crystal structures of KRAS G12X mutants display only minor differences, but the lack of structural differences does not exclude the allosteric effect of the mutation [56]. The shift in the dynamics by G12X is able to occur via the closest hydrophobic hub V14. As the wild-type KRAS has a flexible glycine residue in this position, a G12X mutation alters the dynamics of the neighboring residue A11 or the whole P-loop (including both A11 and V14). As this hydrophobic hub V14 is connected to a hydrophobic network, a local alteration in KRAS dynamics can be conveyed via the hydrophobic network to the other remote structural regions in KRAS in an allosteric manner.

Supporting the fact that V14 is an important hub in the KRAS hydrophobic interaction network, a mutation in this position, V14I, is found to be one of the responsible mutations for the Noonan syndrome [57,58] and may also predispose tumor development [59]. Whereas the V14I mutation does not change the GTPase activity of KRAS, it displays similar affinity to RAF1, as does also the G12V mutant [60]. Therefore, a mutation that has an influence in the dynamics of these hydrophobic hub interactions may have a dramatic influence in overall KRAS dynamics and thereby KRAS signaling.

The most altered interaction pattern within the hydrophobic interaction network in all the hubs is observed with the G12A and G12S mutants. Surprisingly, the other mutations (G12C, G12D, G12R and G12V) are not radically different compared to the wild-type, although some alterations in the hub interactions are evident. However, even though a mutant, such as G12D, displays the same interaction frequencies as the wild-type, the characteristics of the interactions may still differ, as the exact characteristics of these interactions, unfortunately, cannot be derived from this analysis, only their frequencies.

To highlight that the alteration in KRAS dynamics is also present with the mutants that display a minor shift in the hydrophobic hub interaction network compared to the wild-type, is the observed variability in the distribution among the metastable states of exceptional importance. The MSM confirmed the indirect effect of the mutation on the switch-region protein conformations and dynamics. As for MSMs one needs to have extended simulation data, we focused on the most important KRAS G12X mutants (G12D, G12R and G12V). In crystal structures these dynamic metastable states are not observed. This is due to the fact that in the structures the switch regions are disordered, if there are no crystal contacts to the switches. Based on the MSMs, the G12D mutant follows the dynamics of the wild-type more closely than of the G12R and G12V mutants. Intriguingly, this is in line with the findings of the interaction network analysis, where the G12D displayed the most similar profile with the wild-type. In particular, our MD results show that the effects of KRAS G12X mutants are mutation-specific, and suggest that the observed changes in protein conformations and dynamics may alter protein activity [61].

We consider that the difference in the mutant dynamics, for instance the G12V dynamics with its inability to reach the metastable states I and V (Fig 5), may reflect the differences observed in the RAS effector protein binding [8,10]. In fact, simple protein complexes assemble generally via a single pathway [62], and the observed metastable states may correspond to the first steps in the effector protein binding process. These states may be important for specific effector protein binding and pathway activation. However, based on the simulation data we were unable to distinguish if a putative effector protein(s) or a particular signaling pathway(s) is related to a specific metastable state. It needs to be clarified, if these states act as intermediate steps in the KRAS–effector protein association and play a role in the macromolecular recognition process, effecting the association kinetics of the complex formation [63]. Furthermore, multiple other aspects related to the altered dynamics may also affect KRAS mediated signaling. The altered dynamics may cause a conformational change of KRAS on the membrane, resulting in occluded conformation from a specific effector [23], alter the dimer formation [64], or affect KRAS nanoclustering [65]. Furthermore, the altered dynamics may affect the stability of a KRAS–effector complex itself that may lead to a more stable complex, resulting in binding with a longer lifetime, or conversely to a more unstable complex, resulting in faster dissociation. Altogether, this implies that the altered protein dynamics has an influence on the KRAS binding partner selection.

As a crucial factor for KRAS dimerization, the intermolecular D154–R161 salt-bridges between the dimers were recently identified [66]. The KRAS dimerization is a GTP-dependent process [67]. Here we observed that with the GTP-bound wild-type, there is a shift in the dynamics of the putative α4-α5 dimer interface region, manifested by the more unstable intramolecular D154–R161 salt-bridge. This suggests that the more stable intramolecular salt-bridge within the monomer residues could hinder the dimer formation in GDP-bound KRAS, whereas due to the change in the dynamics on the site in GTP-bound KRAS, the more unstable intramolecular salt-bridge could promote the formation of intermolecular salt-bridges among these residues, and thus dimer formation. This intramolecular salt-bridge is again, regardless of the bound ligand, heavily distorted with the G12A and G12S mutants that have a major effect on the hydrophobic interaction networks.

In general, the mutational frequency data combined with the observation from the simulations suggest that at least in the pancreatic cancer, where a KRAS mutation is a key initiator [68], a major shift in KRAS dynamics is not tolerated. This fact is manifested by the low frequencies of G12A and G12S mutants in the pancreas. Moreover, the distorted dynamics could also offer an explanation why the G12S mutant is rarely observed even though it is a transition mutation.

It has been clearly shown that the KRAS G12X mutation has an effect on the intrinsic GTPase activity and that it causes insensitivity for GAPs. However, it seems that the interpretation of the mutation effect on the oncoprotein’s behavior has been oversimplified. First, in specific tissues G12X mutation frequencies exhibit an inexplicable individual bias. Furthermore, the mutation inflicts individual changes in the protein dynamics, affecting the allosteric communication network that conveys the shift in dynamics to the remote sites within KRAS. Finally, this shift in protein dynamics may lead to modulated KRAS mediated signal transduction. We therefore suggest that altered dynamics among KRAS G12X mutants may promote the observed non-random frequencies in specific tissues. In order to establish successful therapies against mutant KRAS-harboring tumors, these discrepancies between the G12X mutants need to be reconsidered thoroughly. Concluding, KRAS G12X mutants are not equal, they are unique.

## Materials and methods

### Atomistic models and molecular dynamics simulations

The simulations were conducted using the GROMACS package v. 4.6 with the (all-atom) OPLS-AA force field [69–72]. For the simulations, a high resolution (1.24 Å) truncated (169/188 residues) GDP-bound wild-type KRAS structure (PDB ID: 4OBE) [73] was selected, where most of the HVR is absent (see S1 Fig). Mutant KRAS structures were generated from the wild-type structure using Maestro [74]. For GTP-systems the GDP was replaced with GTP. As a model for GDP and GTP ligands, the default OPLS-AA parameter set was used and the geometry optimization for GDP and GTP was conducted with Gaussian [75], using the Hartree-Fock method and the 6–31+G** basis set. The partial charges for both ligands were derived from the electrostatic potential by performing a RESP fitting procedure with R.E.D. Tools IV [76,77]. Co-crystallized water molecules from the crystal structure were hold intact, with an exception of GTP-systems with one water molecule, which occupied the γ-phosphate binding position to Mg^2+^. Water molecules were described with the TIP3P model [78]. In each system, the protein was solvated in a cubic box (edges at least 1.3 nm from the protein), and the system was neutralized using a physiological ion concentration (140 mM) of K^+^ and Cl^-^ ions. After energy minimization, the system preparation was done in four stages to obtain properly equilibrated and different initial structures for replica simulations (see S1 Table for details). The simulations were performed with periodic boundary conditions in the NpT ensemble. The V-rescale and Parrinello-Rahman methods were used for temperature (310 K) and pressure (1 atm) coupling, respectively [79,80]. The default 2 fs time step was used for integration of equations of motion. To preserve the length of all bonds, the LINCS algorithm was used [81]. For Lennard-Jones interactions and the real-space part of the particle mesh Ewald electrostatics, a cutoff of 1.0 nm was used [82]. Each system was simulated for 2 μs with 5–10 independent replicas, such that the individual system simulation time was 10–20 μs and the total simulation time was 170 μs.

### Analysis of simulation data

The RMSF were calculated using GROMACS tools [69]. Principal component analysis (PCA) was conducted for the backbone atoms by the GROMACS covariance analysis tools. For PCA, we discarded the first 300 ns and used only the last 1.7 μs of each simulated system, to remove the potential bias of the starting GDP crystal structure from the results. To reduce noise from the flexible terminal regions, we excluded from the analysis the first three residues from the N-terminal and the last five residues from the C-terminal. The PCA structures (Fig 2, S5–S9 Figs) were obtained utilizing the GROMACS tool gmx_anaeig, and visualized with PyMOL 1.8 [83] using a pymol-script Modevectors [84].

The analysis of KRAS interaction networks (the hydrophobic clusters and the salt-bridges) was conducted with PyInteraph [44] and visualized with PyMOL [83]. This analysis was conducted for full trajectories and all residues were included in the analysis.

Markov state model generation was conducted with PyEMMA 2, following the general recommendations [46]. As an input, we used distances between the residues 12, 32, 34, 36, 48, 59, 62, 64, 67, 105, 122, 126, and 138 from the simulation trajectories. We selected these residues based on their functional importance in KRAS (location in the interaction surface with the effector proteins), the results of PCA (dynamical importance), or both. Furthermore, a slow linear subspace from this input was estimated by TICA [85], as TICA highlights the slowest motions from simulations and is highly suitable for generation of a MSM [86], using 40 ns as a lag-time, and two dimensions. Furthermore, the output of TICA was clustered using the k-means clustering, and the discretized trajectories from the clustering analysis were used to generate the BayesianMSM. The number of clusters in k-means were set as √N, as recommended in [46]. The microstates were grouped in seven metastable states by the Perron-cluster cluster analysis (PCCA++) method [87], based on the spectral analysis (S13C Fig). The generated models were validated by two methods. First, we calculated the resulting timescales and found that the timescales were constant in the used 40 ns lag-time (S13–S15 Figs). Furthermore, we conducted the Chapman-Kolmogorov test, which displayed that the model followed the expected estimates. The occupations of individual mutants in each metastable state (Fig 5) were computed from their individual Markov models.

### Statistical analyses of mutation frequencies

The *KRAS* G12X mutation data was collected from COSMIC database v.79 (http://cancer.sanger.ac.uk/cosmic/) [2]. In our assessment of the *KRAS* G12X mutation probability, we included only single nucleotide substitutions. This choice was made based on the fact that more complex mutations and their probabilities (e.g., adjacent double substitutions) are not predictable with the existing knowledge. Fisher’s exact test was used to analyze the specific differences in mutation frequencies.

## Supporting information

S1 Fig (**A**) Regardless of the bound effector protein, the RAS conformation in the HRAS–effector protein complexes remain identical. The superposed RAS structures have been taken from RAS-complexes as follows: RalGDS (PDB ID 4G0N), Raf-1 (PDB ID 1LFD) and PI3Kγ (PDB ID 1HE8). Highlighted in the figure are the switch-I (red) and switch-II (blue) regions, residue 12 (orange), and GNP-ligand (cyan ball and sticks). (**B**) The effector proteins bind RAS on top of the switch regions, indicating no direct contact between residue 12 (orange) and effector proteins. Shown is the RAS–PI3Kγ complex (PDB ID 1HE8), with PI3Kγ identified (teal surface). The RAS surface is colored as in panel **A**, and GNP represented as CPK. (**C**) The effector protein-binding interface is identical with HRAS and KRAS. The hypervariable region (yellow) is not present in crystal structures (**A** and **B**). (**D**) The locations of the secondary structural elements in KRAS. Also, the positions of the Cα of residue 12 (cyan sphere), P-loop (cyan), γ-phosphate of the GTP (ball & stick), and the SAK-motif (black) are highlighted.
(TIF)

S2 Fig (**A**) Distributions of *KRAS* G12X mutations calculated based on an assumption of random events dictated by a value of 2.3 for the transition:transversion ratio. The occurrence of mutations related to random mutation frequencies in (**B**) all tissues, (**C**) the pancreas, (**D**) the large intestine, (**E**) the lung, (**F**) the peritoneum, (**G**) the small intestine, (**H**) the biliary tract, (**I**) the endometrium, and (**j**) the ovary. Individual tissues (**B**-**J**) have been arranged in decreasing significance for the overall G12X mutation frequency (%), **B** displaying the highest and **J** the lowest. Mutation data have been collected from the COSMIC database v.79.
(TIF)

S3 Fig Root-mean-square fluctuation (RMSF) of GDP-bound systems: (**A**) G12A, (**B**) G12C, (**C**) G12D, (**D**) G12R, (**E**) G12S, (**F**) G12V, and (**G**) wild-type. Error bars indicate the standard error (SE). The average of all GDP-bound systems is indicated with an orange line. RMSF has been calculated over a period of 300–2000 ns, with switch-I (residues 30–40) and switch-II (residues 58–72) identified as given here.
(TIF)

S4 Fig Root-mean-square fluctuation (RMSF) of GTP-bound systems: (**A**) G12A, (**B**) G12C, (**C**) G12D, (**D**) G12R, (**E**) G12S, (**F**) G12V, and (**G**) wild-type. Error bars indicate the standard error (SE). The average of all GDP-bound systems is indicated with an orange line. RMSF has been calculated over a period of 300–2000 ns, with switch-I (residues 30–40) and switch-II (residues 58–72) identified as given here.
(TIF)

S5 Fig Extreme movements of principal component 3 (PC3) given by PCA in (**A**) all GDP- and GTP-bound systems. (**B**) The contributions (%) of principal components 1–10 (PC1-PC10). PCA3 vs. PC1 score plots (heat map) of (**C**) GDP-bound and (**D**) and GTP-bound systems. Top-left boxes comprise all the systems with (**C**) GDP or (**D**) GTP. For conformational reference, the backbone conformation of RAS from the RAS–effector and RAS–GEF complexes is included in the plots, where switch-I and switch-II are in a totally closed conformation (blue crosses; from RAS–effector protein complexes) or switch-I is in a fully open conformation (cyan crosses; from a RAS–GEF complex). Reference RAS structures were obtained from HRAS–effector protein complexes (PDB IDs: 1HE8, 1LFD, 4G0N) and from the HRAS–Sos complex (PDB ID: 4NYJ).
(TIF)

S6 Fig Extreme movements of principal component 1 (PC1) given by PCA in (**A**) all GDP-bound systems, and in individual GDP-bound systems: (**B**) G12A, (**C**) G12C, (**D**) G12D, (**E**) G12R, (**F**) G12S, (**G**) G12V, and (**H**) wild-type. Highlighted regions are position of G12X (orange), switch-I (red), and switch-II (blue).
(TIF)

S7 Fig Extreme movements of principal component 2 (PC2) given by PCA in (**A**) all GDP-bound systems, and in individual GDP-bound systems: (**B**) G12A, (**C**) G12C, (**D**) G12D, (**E**) G12R, (**F**) G12S, (**G**) G12V, and (**H**) wild-type. Highlighted regions are position of G12X (orange), switch-I (red), and switch-II (blue).
(TIF)

S8 Fig Extreme movements of principal component 1 (PC1) given by PCA in (**A**) all GTP-bound systems, and in individual GTP-bound systems: (**B**) G12A, (**C**) G12C, (**D**) G12D, (**E**) G12R, (**F**) G12S, (**G**) G12V, and (**H**) wild-type. Highlighted regions are position of G12X (orange), switch-I (red), and switch-II (blue).
(TIF)

S9 Fig Extreme movements of principal component 2 (PC2) given by PCA in (**A**) all GTP-bound systems, and in individual GTP-bound systems: (**B**) G12A, (**C**) G12C, (**D**) G12D, (**E**) G12R, (**F**) G12S, (**G**) G12V, and (**H**) wild-type. Highlighted regions are position of G12X (orange), switch-I (red), and switch-II (blue).
(TIF)

S10 Fig Hydrophobic hub interactions in individual systems. Hydrophobic interactions and their frequencies from hubs: (**A**) V14, (**B**) M72, (**C**) F78, (**D**) L79, (**E**) F90, and (**F**) I100. The hydrophobic interactions that are present (>10%) at least in one system are shown.
(TIF)

S11 Fig Hydrophobic hub interactions and D154-R161 salt-bridge in individual systems. Hydrophobic interactions and their frequencies from hubs: (**A**) V114, (**B**) A146, (**C**) A155, (**D**) F156, and (**E**) L159. The hydrophobic interactions that are present (>10%) at least in one system are shown. (**F**) The intramolecular salt-bridge D154-R161 frequency.
(TIF)

S12 Fig Example of 8oxoG-DNA in complex with N-glycosylase/DNA lyase (*OGG1*). The 8oxoG is oriented inside the catalytic site of N-glycosylase/DNA lyase enzyme. PDB ID: 2NOB.
(TIF)

S13 Fig Validation of Markov state model. (**A**) The resulting timescales are constant with the used lag-time 40 ns. (**B**) The Chapman-Kolmogorov test shows that MSM (black line) follows the observed trajectory (blue dotted line, with estimate error). (C) The spectral analysis revealed large timescale separation between the third and fourth, and sixth and seventh relaxation timescales.
(TIF)

S14 Fig Validation of Markov state model for each system. The resulting timescales are constant with the used lag-time 40 ns for individual MSMs: (**A**) G12D, (**B**) G12R, (**C**) G12V, and (**D**) wild-type.
(TIF)

S15 Fig The Chapman-Kolmogorov test for individual systems’ MSMs with the first six states: (**A**) G12D, (**B**) G12R, (**C**) G12V, and (**D**) wild-type. Shown are data for MSM (black line) and the observed trajectory (blue dotted line, with estimated error).
(TIF)

S1 Table List of simulation systems showing their simulation times, number of repeats carried out for each system, number of atoms in each system, and numbers of counterions used for neutralization. (PDF)
