# Supplementary figures and images for: Assessment of mutation probabilities of KRAS G12 missense mutants and their long-timescale dynamics by atomistic molecular simulations and Markov state modeling
Source: PLoS Comput Biol. 2018 Sep 10;14(9):e1006458. doi: 10.1371/journal.pcbi.1006458 (PMC6147662; doi:10.1371/journal.pcbi.1006458)

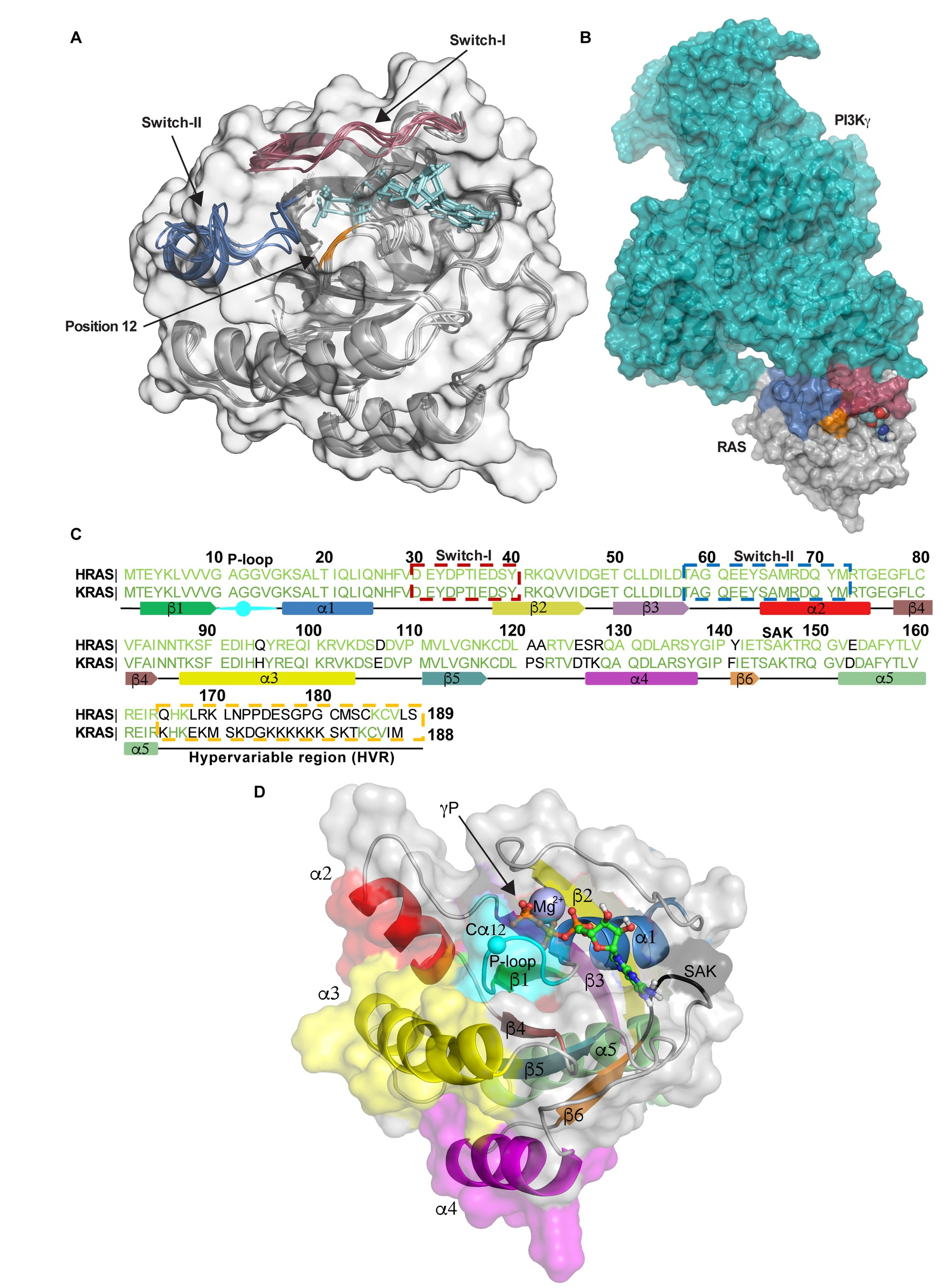

Supplement: S1 Fig — (A) Regardless of the bound effector protein, the RAS conformation in the HRAS–effector protein complexes remain identical. The superposed RAS structures have been taken from RAS-complexes as follows: RalGDS (PDB ID 4G0N), Raf-1 (PDB ID 1LFD) and PI3Kγ (PDB ID 1HE8). Highlighted in the figure are the switch-I (red) and switch-II (blue) regions, residue 12 (orange), and GNP-ligand (cyan ball and sticks). (B) The effector proteins bind RAS on top of the switch regions, indicating no direct contact between residue 12 (orange) and effector proteins. Shown is the RAS–PI3Kγ complex (PDB ID 1HE8), with PI3Kγ identified (teal surface). The RAS surface is colored as in panel A, and GNP represented as CPK. (C) The effector protein-binding interface is identical with HRAS and KRAS. The hypervariable region (yellow) is not present in crystal structures (A and B). (D) The locations of the secondary structural elements in KRAS. Also, the positions of the Cα of residue 12 (cyan sphere), P-loop (cyan), γ-phosphate of the GTP (ball & stick), and the SAK-motif (black) are highlighted. (TIF) [file pcbi.1006458.s001.tif]

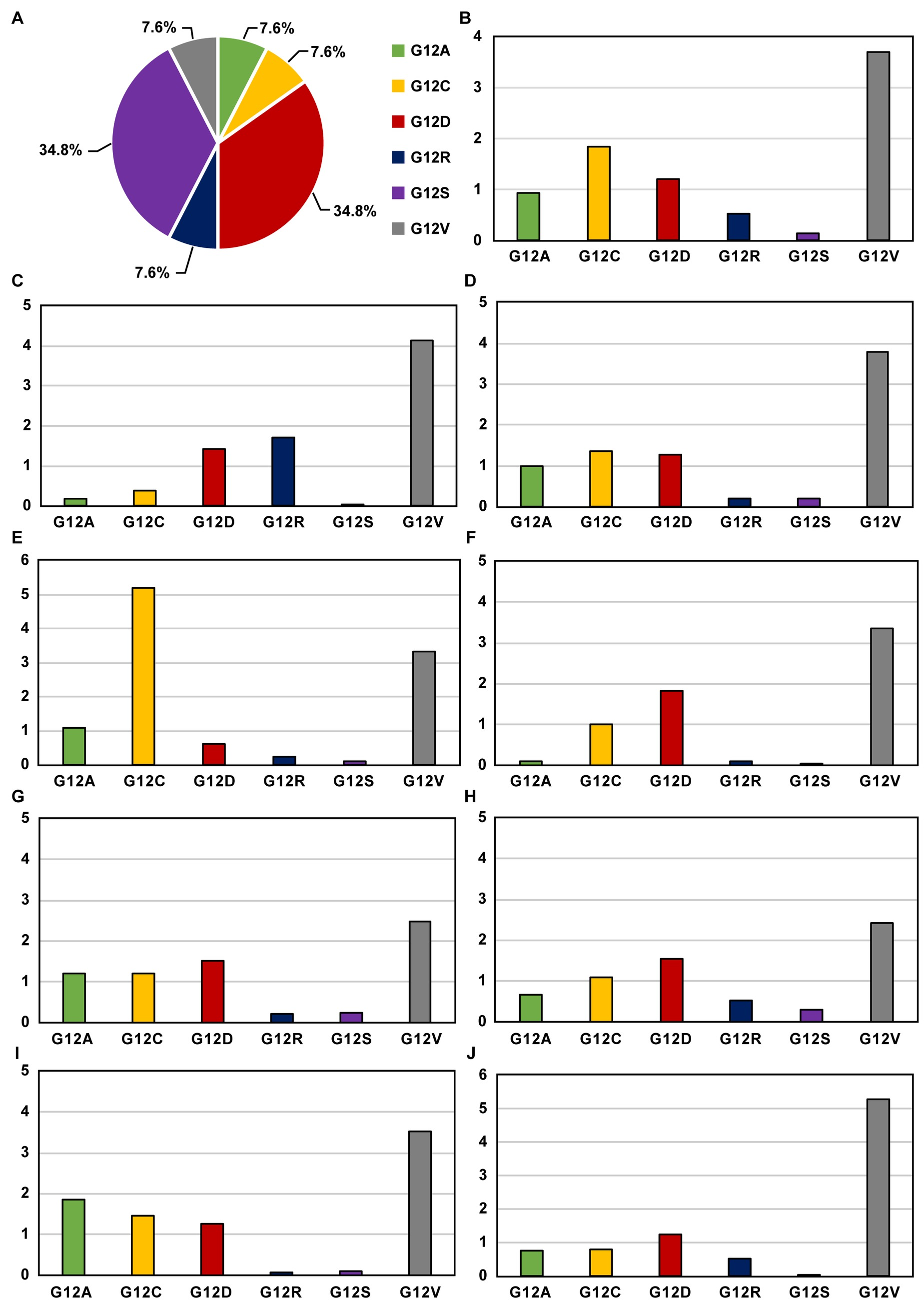

Supplement: S2 Fig — (A) Distributions of KRAS G12X mutations calculated based on an assumption of random events dictated by a value of 2.3 for the transition:transversion ratio. The occurrence of mutations related to random mutation frequencies in (B) all tissues, (C) the pancreas, (D) the large intestine, (E) the lung, (F) the peritoneum, (G) the small intestine, (H) the biliary tract, (I) the endometrium, and (j) the ovary. Individual tissues (B-J) have been arranged in decreasing significance for the overall G12X mutation frequency (%), B displaying the highest and J the lowest. Mutation data have been collected from the COSMIC database v.79. (TIF) [file pcbi.1006458.s002.tif]

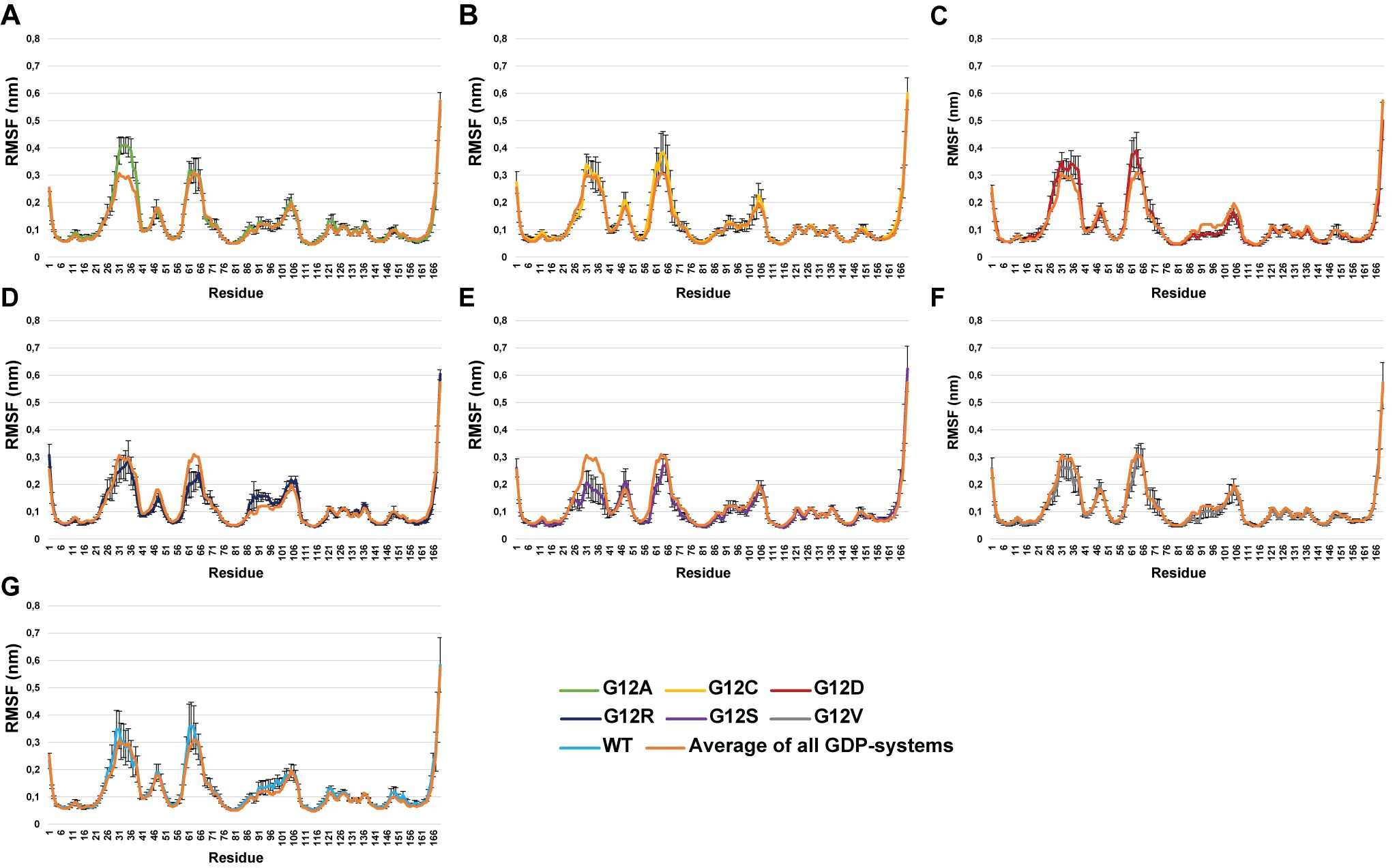

Supplement: S3 Fig — Root-mean-square fluctuation (RMSF) of GDP-bound systems: (A) G12A, (B) G12C, (C) G12D, (D) G12R, (E) G12S, (F) G12V, and (G) wild-type. Error bars indicate the standard error (SE). The average of all GDP-bound systems is indicated with an orange line. RMSF has been calculated over a period of 300–2000 ns, with switch-I (residues 30–40) and switch-II (residues 58–72) identified as given here. (TIF) [file pcbi.1006458.s003.tif]

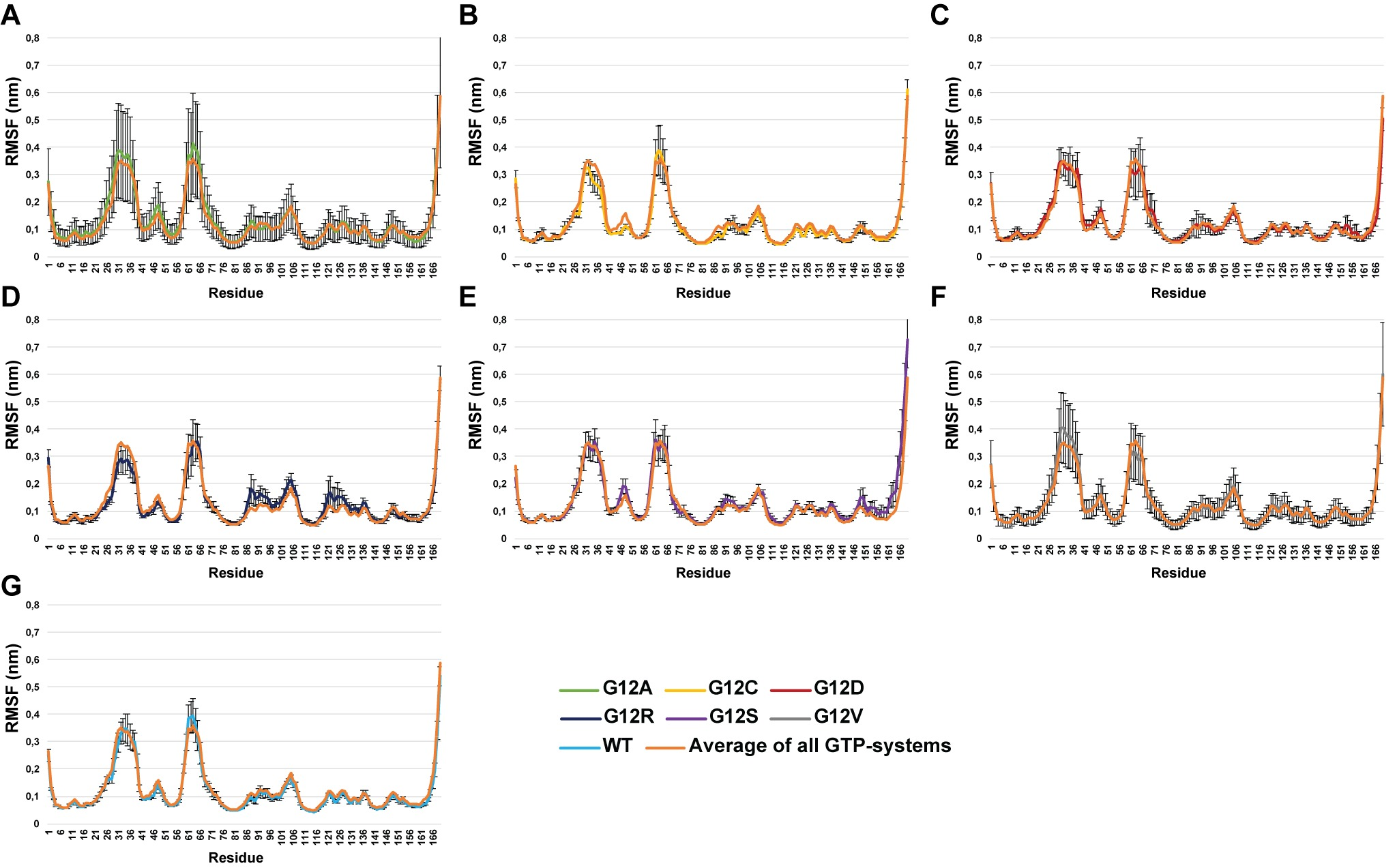

Supplement: S4 Fig — Root-mean-square fluctuation (RMSF) of GTP-bound systems: (A) G12A, (B) G12C, (C) G12D, (D) G12R, (E) G12S, (F) G12V, and (G) wild-type. Error bars indicate the standard error (SE). The average of all GDP-bound systems is indicated with an orange line. RMSF has been calculated over a period of 300–2000 ns, with switch-I (residues 30–40) and switch-II (residues 58–72) identified as given here. (TIF) [file pcbi.1006458.s004.tif]

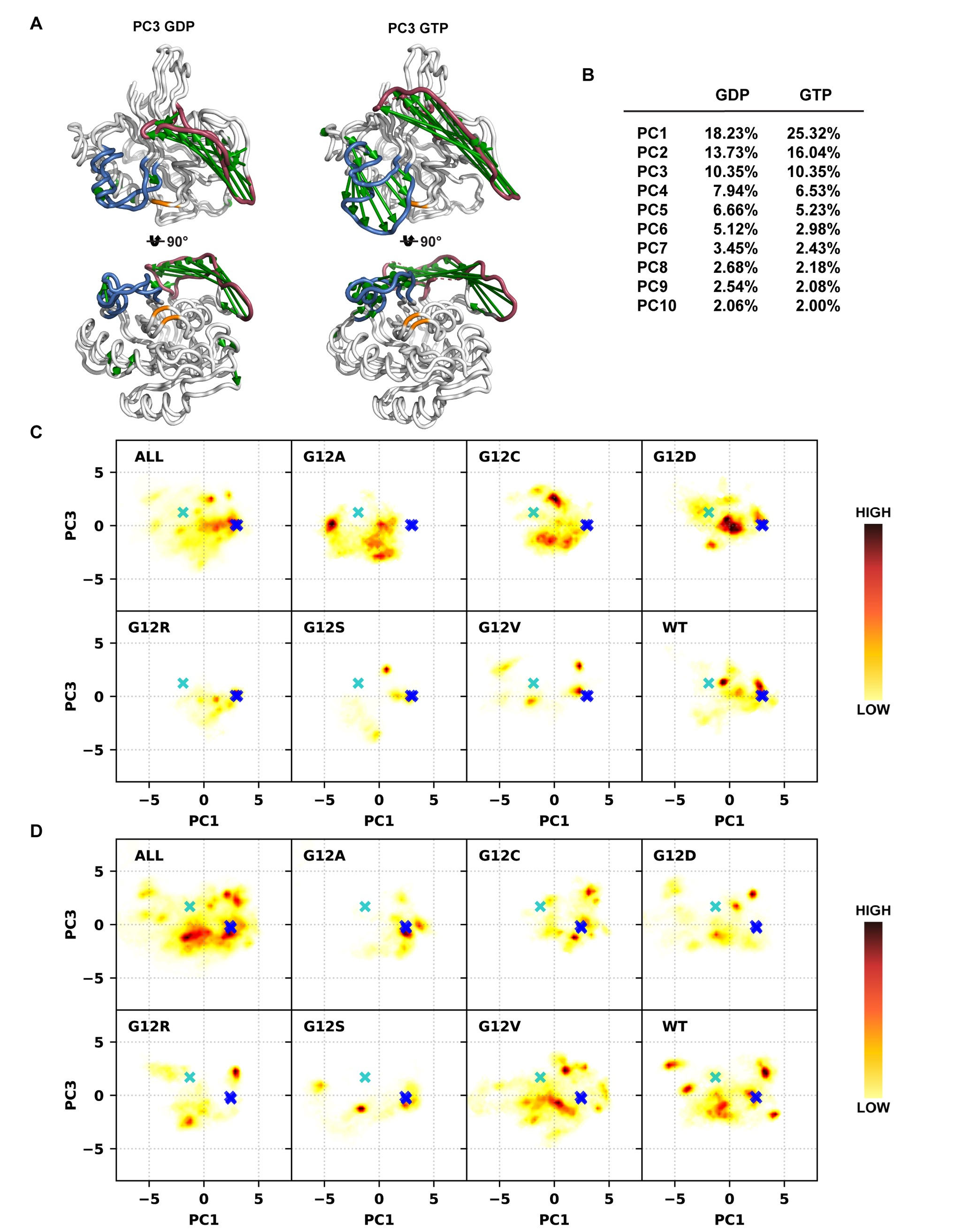

Supplement: S5 Fig — Extreme movements of principal component 3 (PC3) given by PCA in (A) all GDP- and GTP-bound systems. (B) The contributions (%) of principal components 1–10 (PC1-PC10). PCA3 vs. PC1 score plots (heat map) of (C) GDP-bound and (D) and GTP-bound systems. Top-left boxes comprise all the systems with (C) GDP or (D) GTP. For conformational reference, the backbone conformation of RAS from the RAS–effector and RAS–GEF complexes is included in the plots, where switch-I and switch-II are in a totally closed conformation (blue crosses; from RAS–effector protein complexes) or switch-I is in a fully open conformation (cyan crosses; from a RAS–GEF complex). Reference RAS structures were obtained from HRAS–effector protein complexes (PDB IDs: 1HE8, 1LFD, 4G0N) and from the HRAS–Sos complex (PDB ID: 4NYJ). (TIF) [file pcbi.1006458.s005.tif]

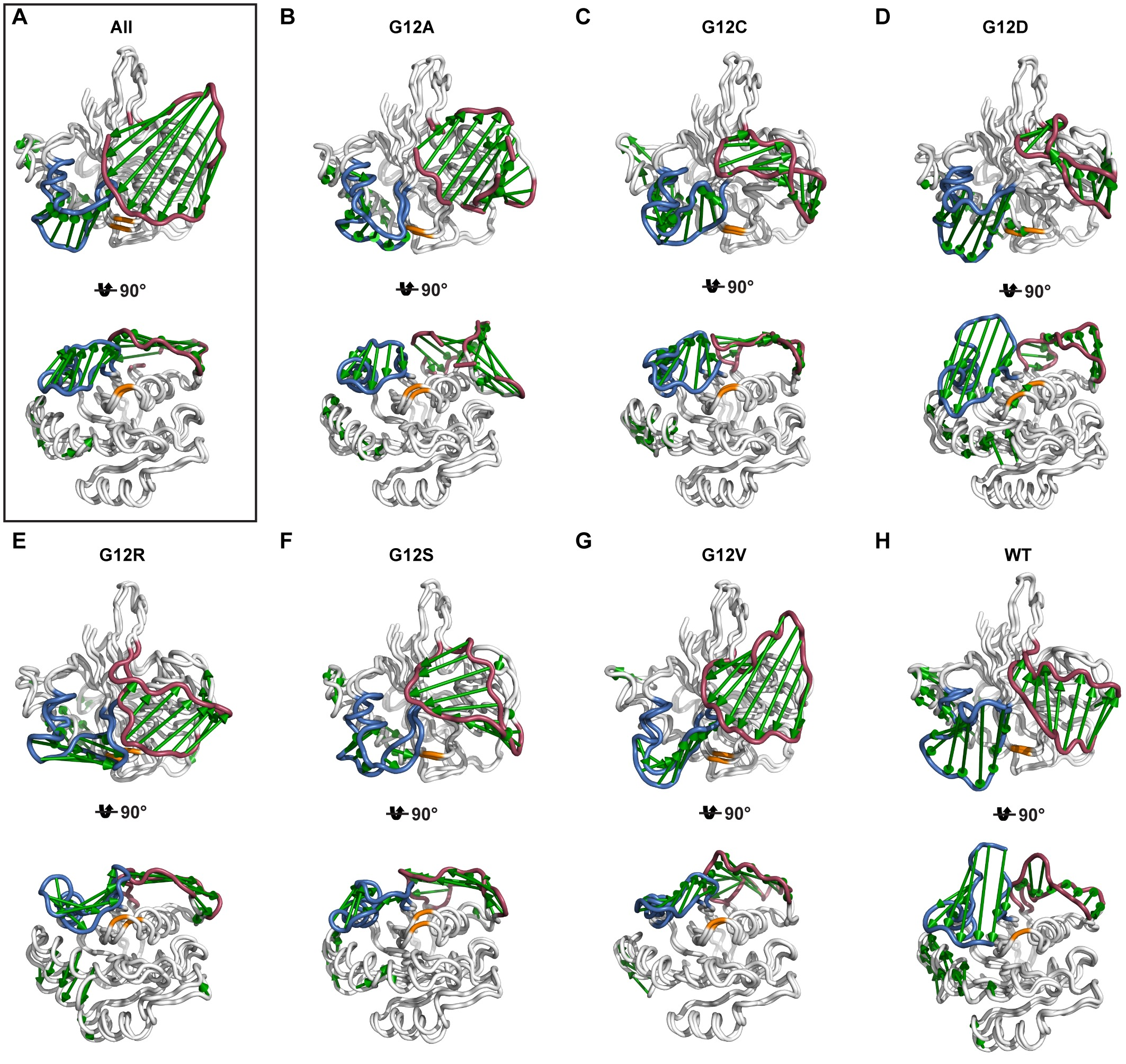

Supplement: S6 Fig — Extreme movements of principal component 1 (PC1) given by PCA in (A) all GDP-bound systems, and in individual GDP-bound systems: (B) G12A, (C) G12C, (D) G12D, (E) G12R, (F) G12S, (G) G12V, and (H) wild-type. Highlighted regions are position of G12X (orange), switch-I (red), and switch-II (blue). (TIF) [file pcbi.1006458.s006.tif]

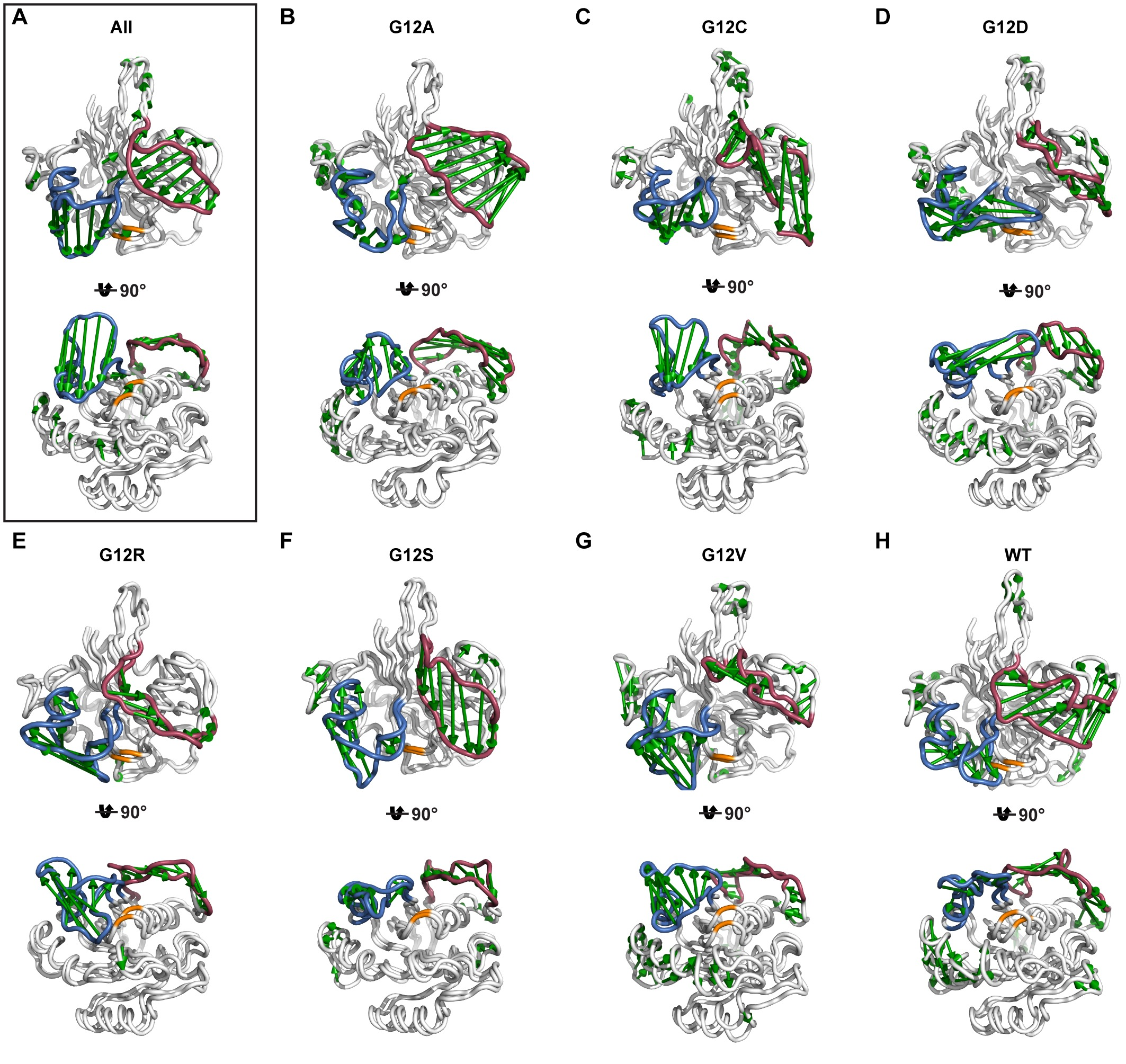

Supplement: S7 Fig — Extreme movements of principal component 2 (PC2) given by PCA in (A) all GDP-bound systems, and in individual GDP-bound systems: (B) G12A, (C) G12C, (D) G12D, (E) G12R, (F) G12S, (G) G12V, and (H) wild-type. Highlighted regions are position of G12X (orange), switch-I (red), and switch-II (blue). (TIF) [file pcbi.1006458.s007.tif]

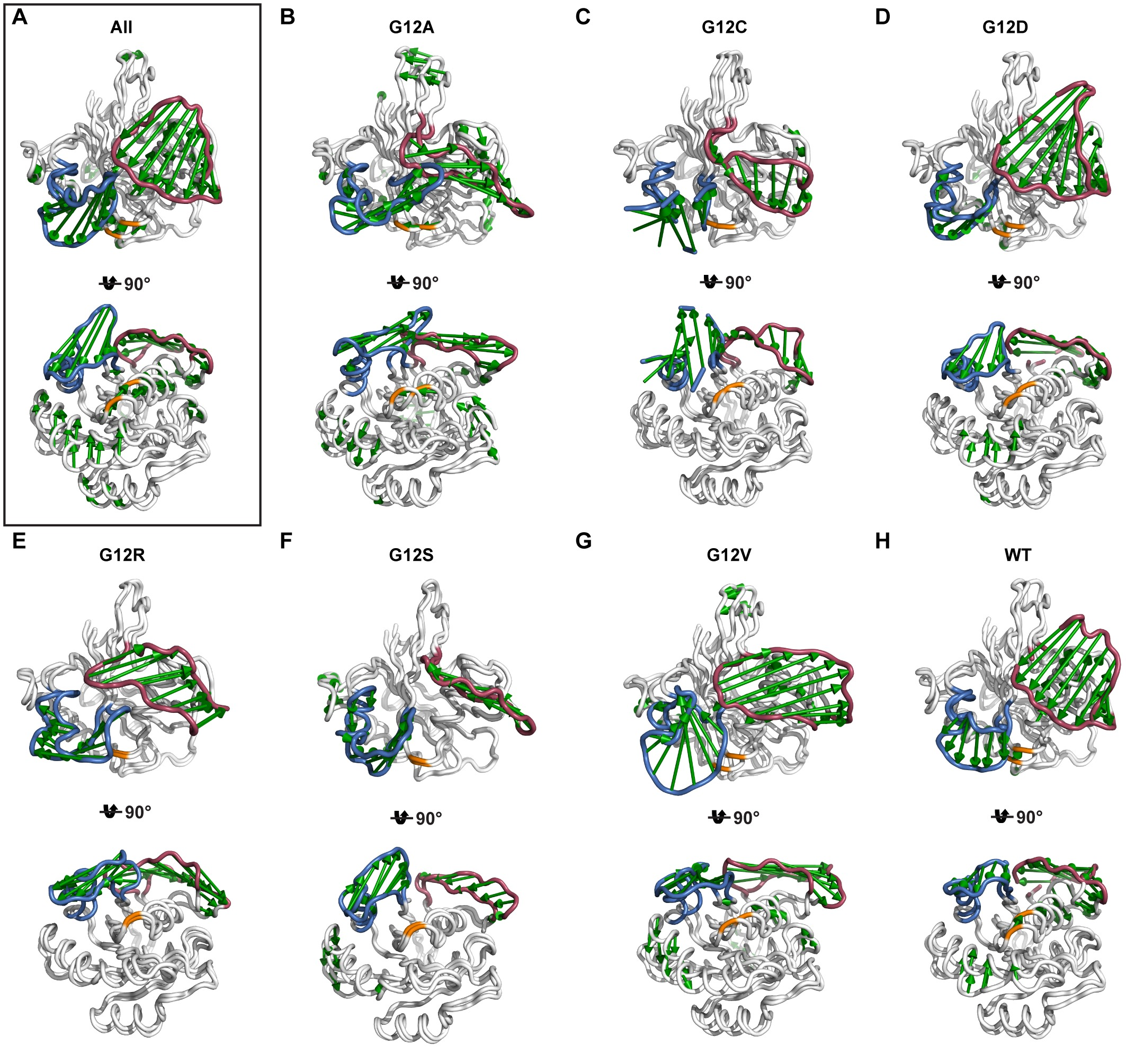

Supplement: S8 Fig — Extreme movements of principal component 1 (PC1) given by PCA in (A) all GTP-bound systems, and in individual GTP-bound systems: (B) G12A, (C) G12C, (D) G12D, (E) G12R, (F) G12S, (G) G12V, and (H) wild-type. Highlighted regions are position of G12X (orange), switch-I (red), and switch-II (blue). (TIF) [file pcbi.1006458.s008.tif]

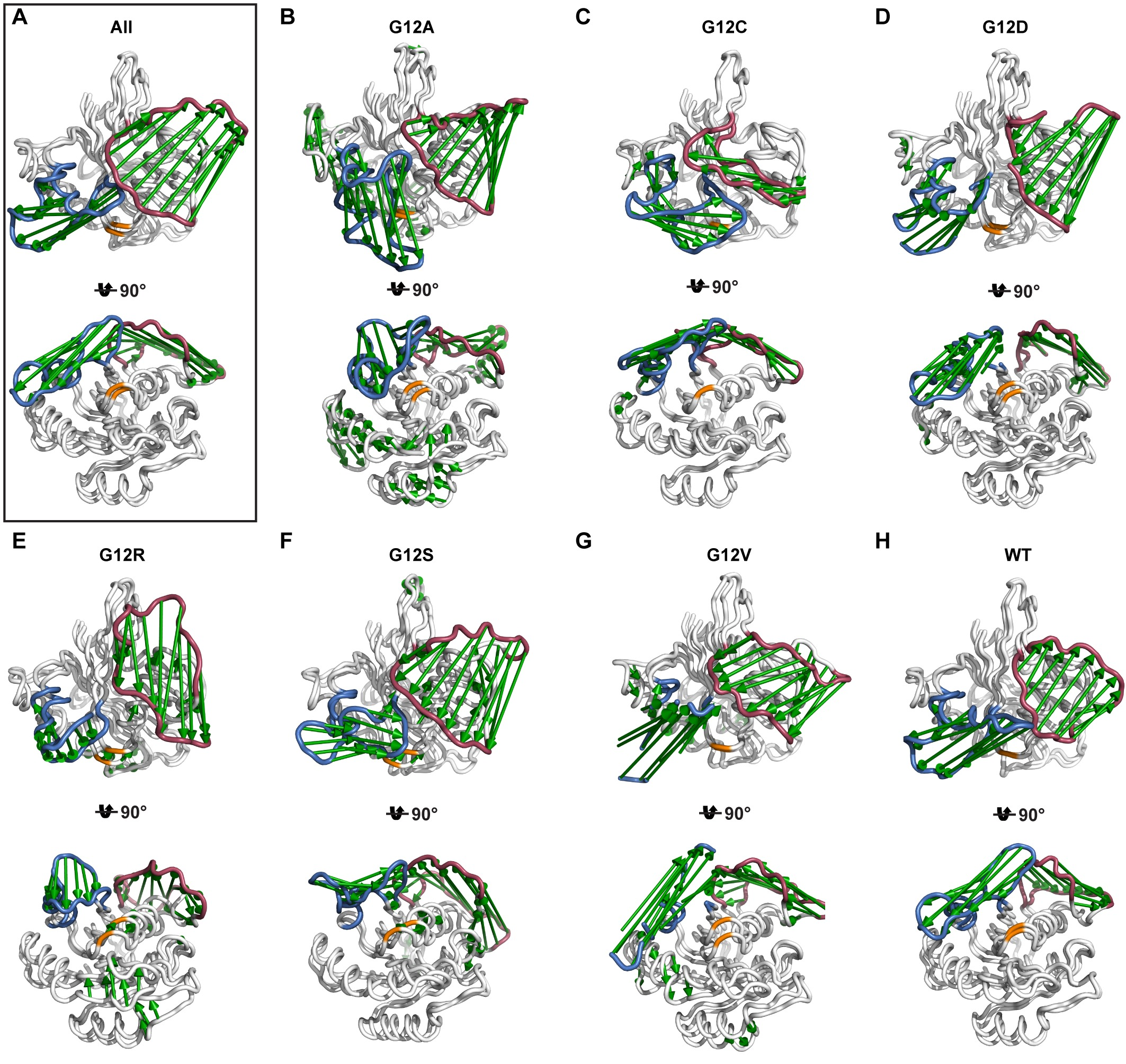

Supplement: S9 Fig — Extreme movements of principal component 2 (PC2) given by PCA in (A) all GTP-bound systems, and in individual GTP-bound systems: (B) G12A, (C) G12C, (D) G12D, (E) G12R, (F) G12S, (G) G12V, and (H) wild-type. Highlighted regions are position of G12X (orange), switch-I (red), and switch-II (blue). (TIF) [file pcbi.1006458.s009.tif]

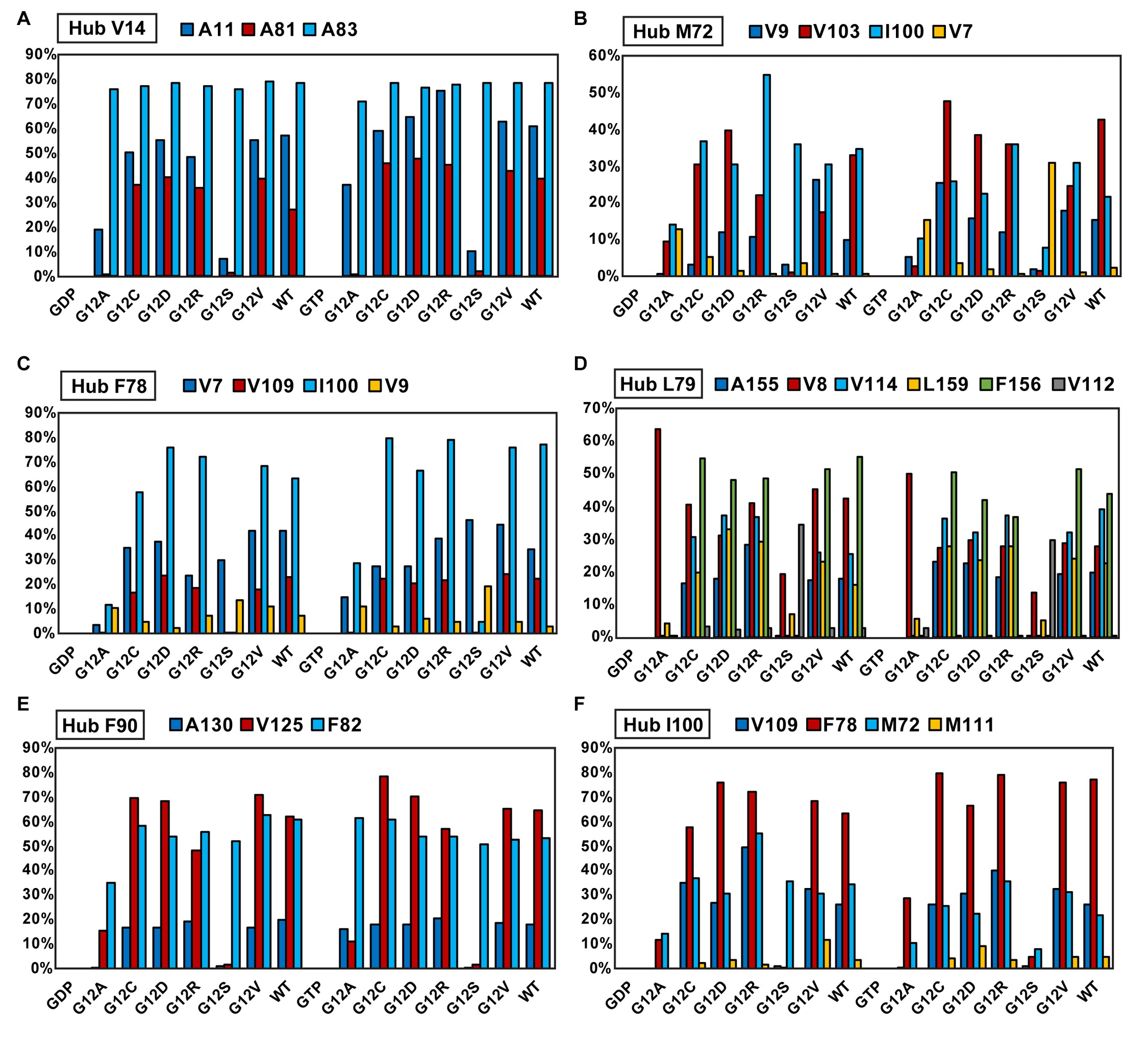

Supplement: S10 Fig — Hydrophobic interactions and their frequencies from hubs: (A) V14, (B) M72, (C) F78, (D) L79, (E) F90, and (F) I100. The hydrophobic interactions that are present (>10%) at least in one system are shown. (TIF) [file pcbi.1006458.s010.tif]

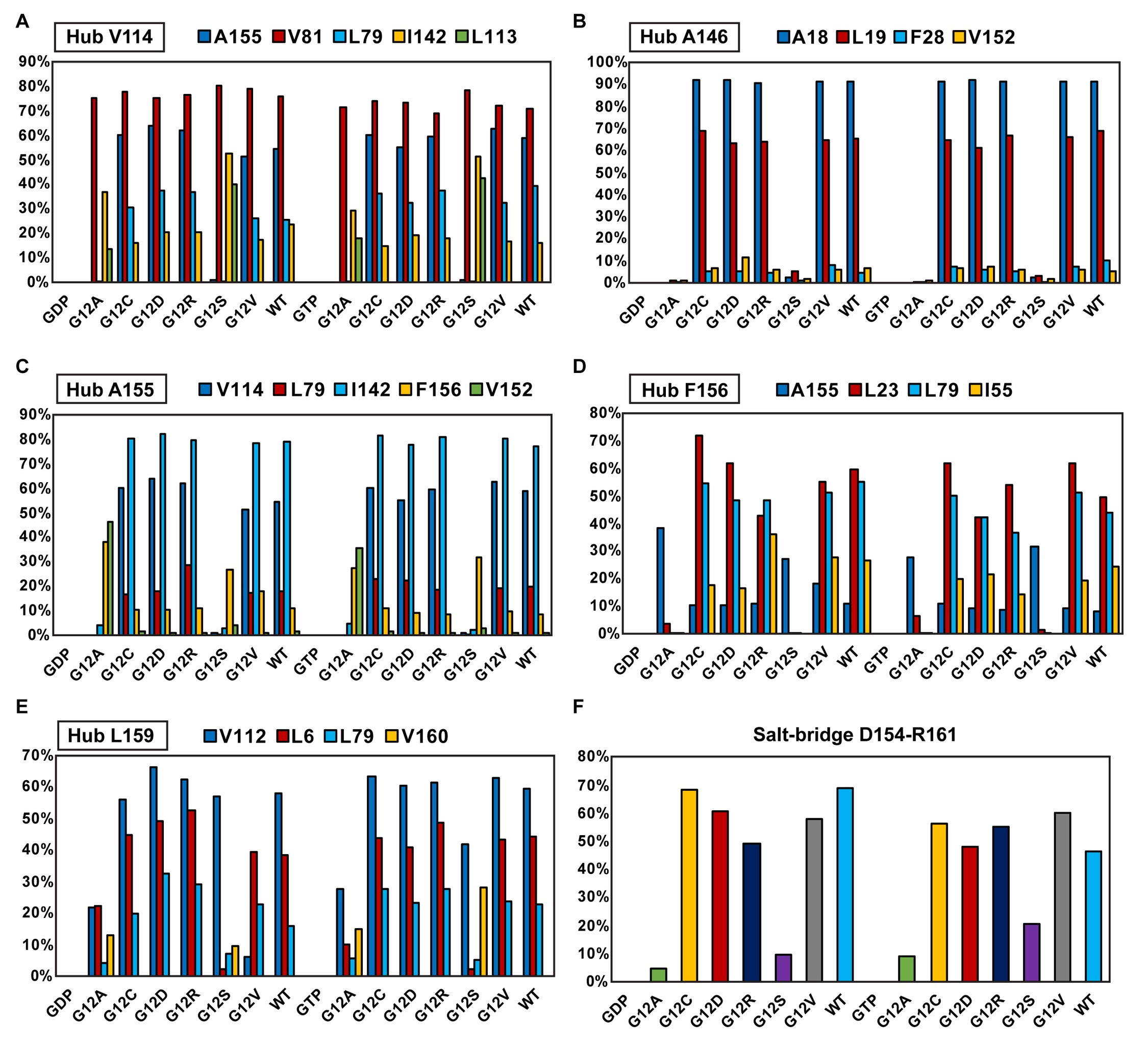

Supplement: S11 Fig — Hydrophobic interactions and their frequencies from hubs: (A) V114, (B) A146, (C) A155, (D) F156, and (E) L159. The hydrophobic interactions that are present (>10%) at least in one system are shown. (F) The intramolecular salt-bridge D154-R161 frequency. (TIF) [file pcbi.1006458.s011.tif]

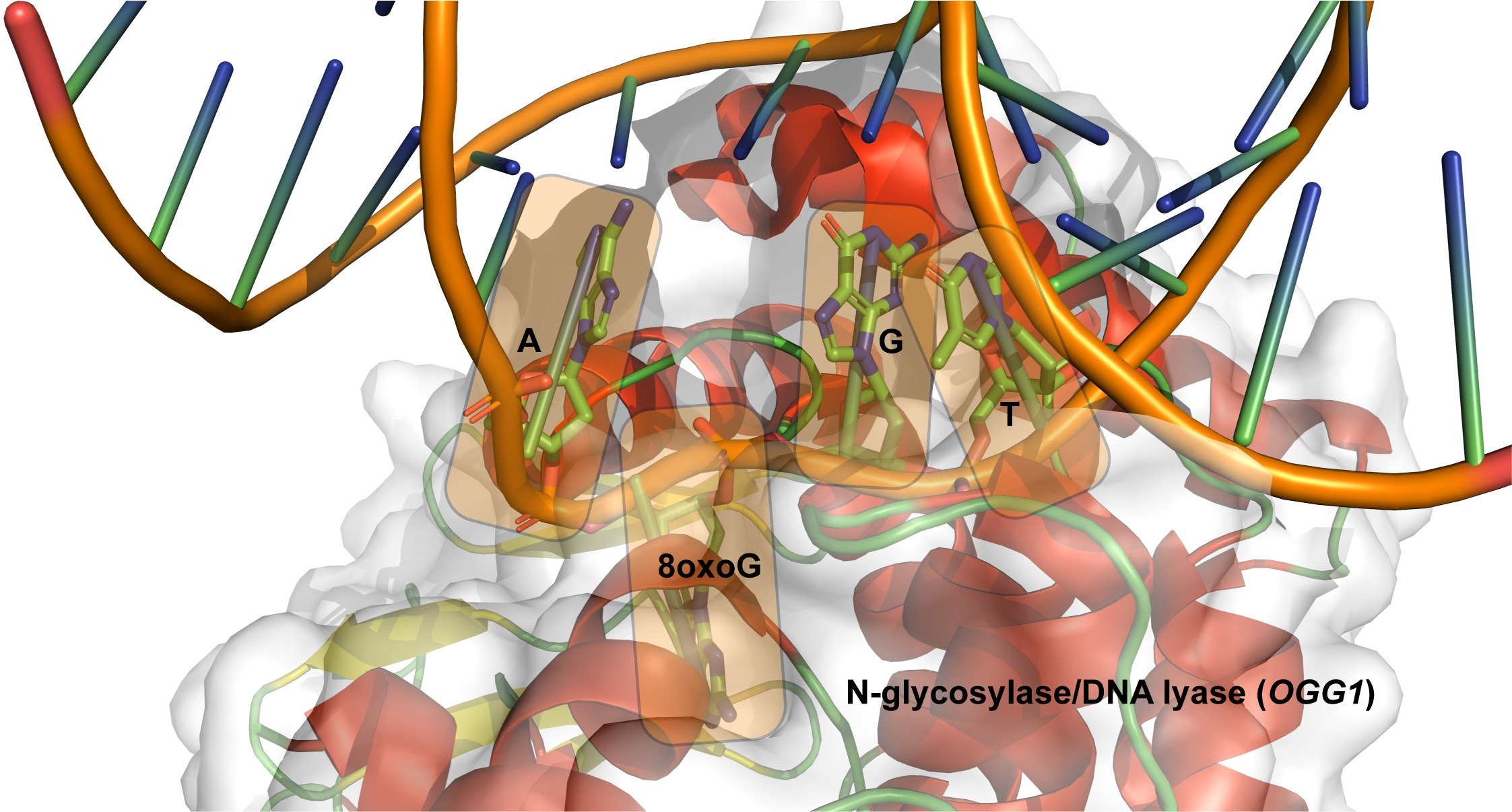

Supplement: S12 Fig — The 8oxoG is oriented inside the catalytic site of N-glycosylase/DNA lyase enzyme. PDB ID: 2NOB. (TIF) [file pcbi.1006458.s012.tif]

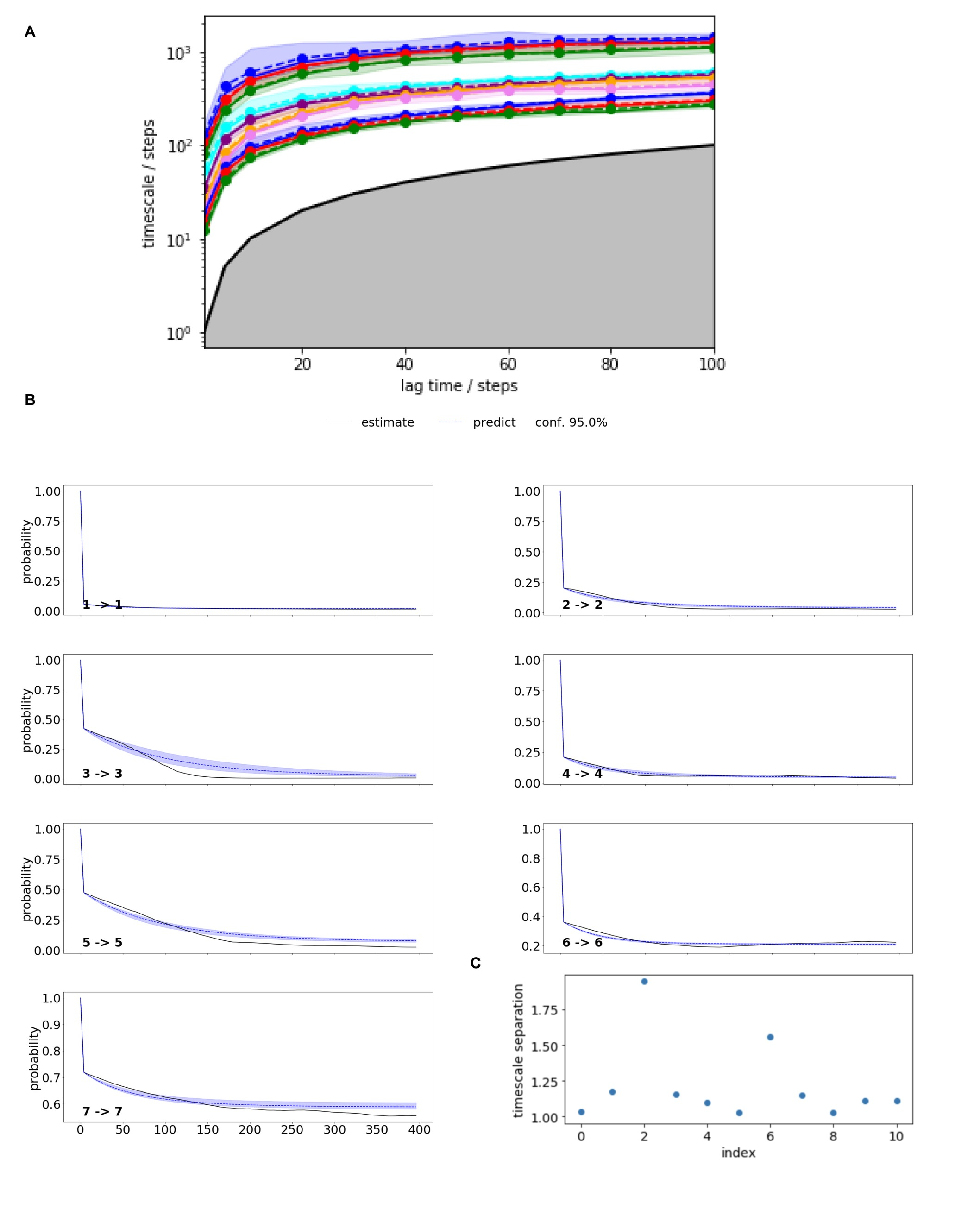

Supplement: S13 Fig — (A) The resulting timescales are constant with the used lag-time 40 ns. (B) The Chapman-Kolmogorov test shows that MSM (black line) follows the observed trajectory (blue dotted line, with estimate error). (C) The spectral analysis revealed large timescale separation between the third and fourth, and sixth and seventh relaxation timescales. (TIF) [file pcbi.1006458.s013.tif]

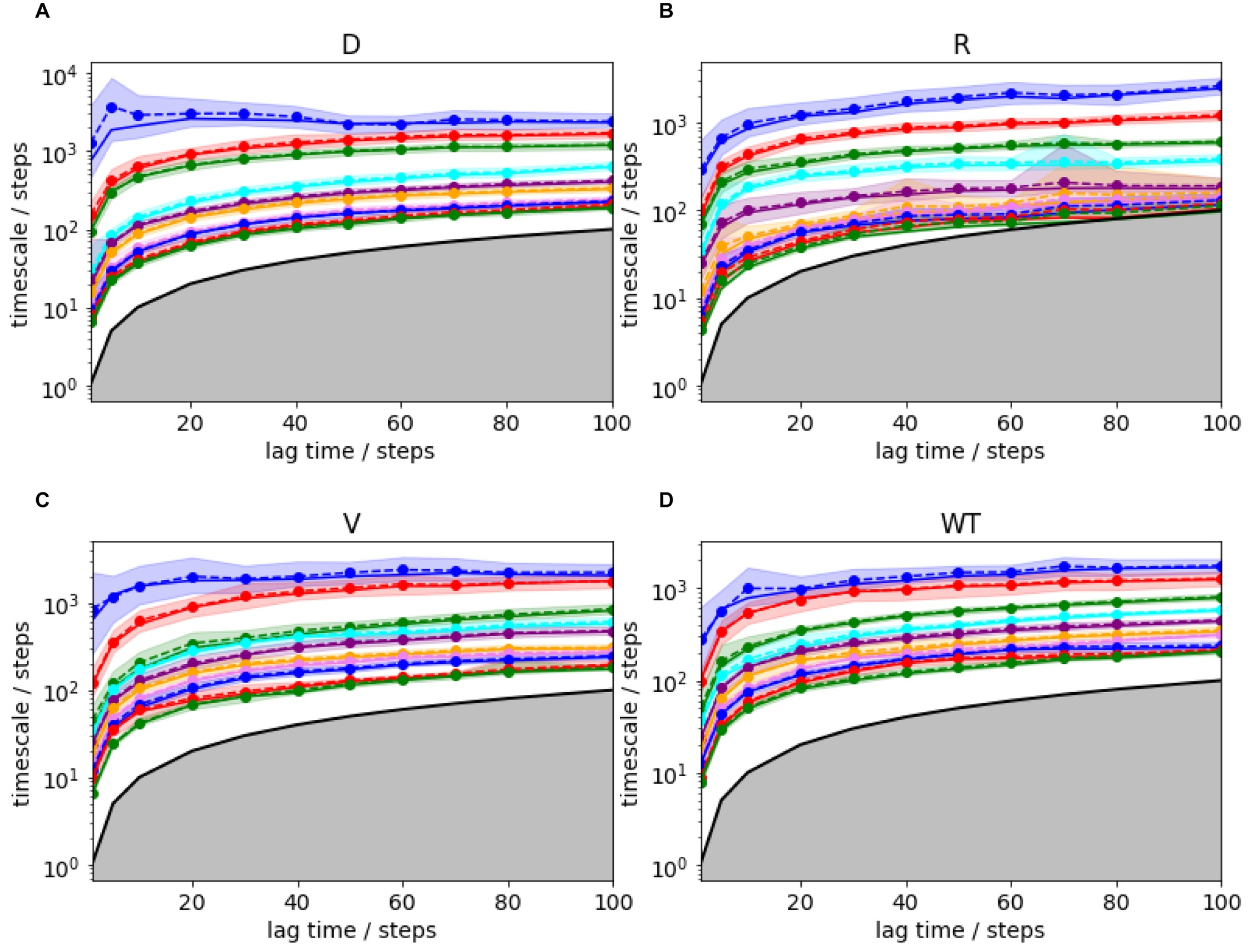

Supplement: S14 Fig — The resulting timescales are constant with the used lag-time 40 ns for individual MSMs: (A) G12D, (B) G12R, (C) G12V, and (D) wild-type. (TIF) [file pcbi.1006458.s014.tif]

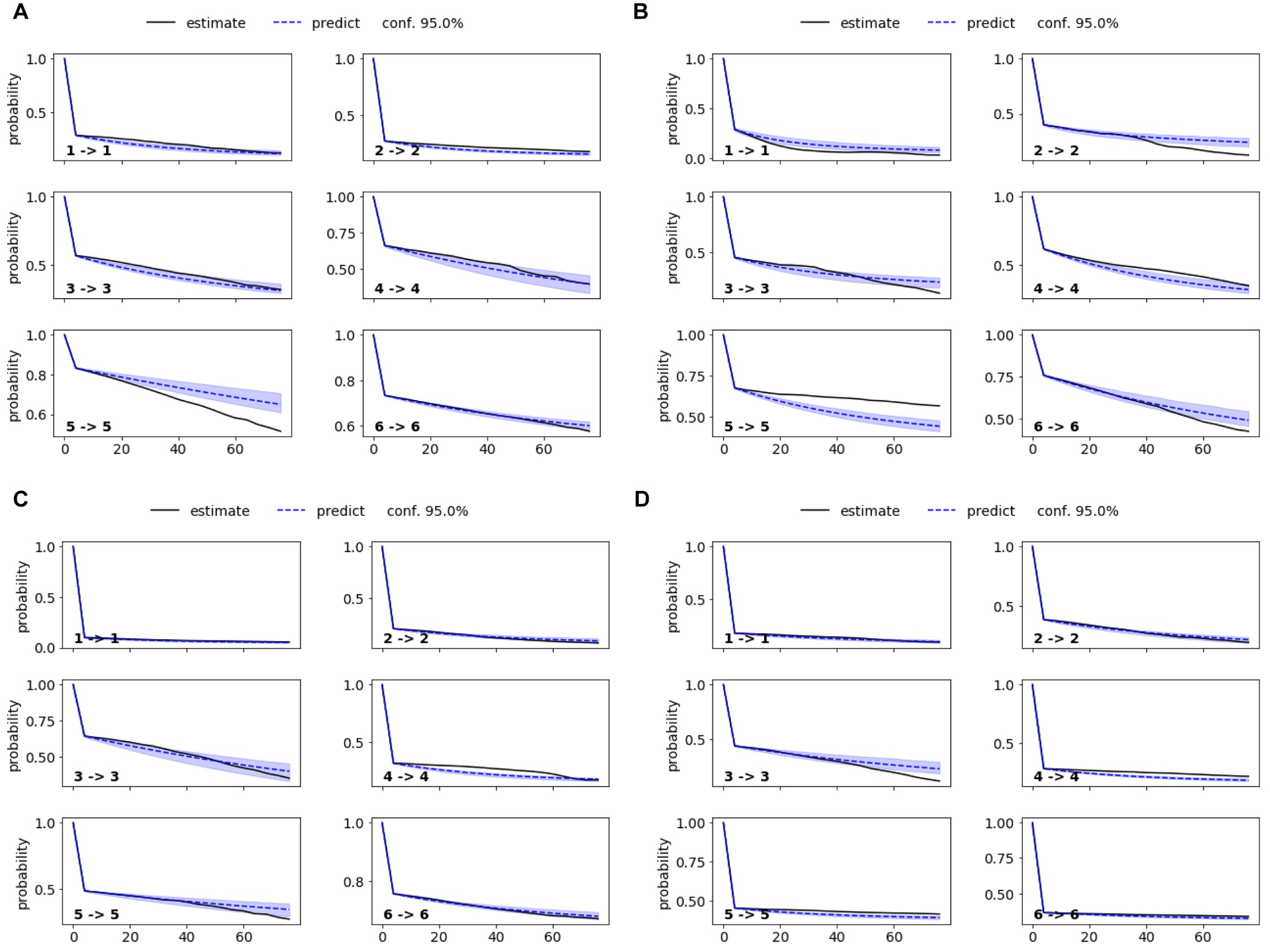

Supplement: S15 Fig — The Chapman-Kolmogorov test for individual systems’ MSMs with the first six states: (A) G12D, (B) G12R, (C) G12V, and (D) wild-type. Shown are data for MSM (black line) and the observed trajectory (blue dotted line, with estimated error). (TIF) [file pcbi.1006458.s015.tif]
