# Supplementary material for: Assessment of mutation probabilities of KRAS G12 missense mutants and their long-timescale dynamics by atomistic molecular simulations and Markov state modeling
Source: PLoS Comput Biol. 2018 Sep 10;14(9):e1006458. doi: 10.1371/journal.pcbi.1006458 (PMC6147662; doi:10.1371/journal.pcbi.1006458)
Supplement: S1 Table — (PDF) [file pcbi.1006458.s016.pdf]

S1 Table. List of simulation systems showing their simulation times, number of repeats carried out for each system, number of atoms in each system, and numbers of counterions used for neutralization.

K-Ras	Ligand	Replicas (á 2 µs)*	No. of atoms in the system	No. of K^+ ions	No. of Cl^- ions
G12A	GDP	5	32 741	35	28
G12A	GTP	5	32 743	36	28
G12C	GDP	5	32 739	35	28
G12C	GTP	5	32 741	36	28
G12D	GDP	5	32 741	36	28
G12D	GTP	10	32 743	37	28
G12R	GDP	5	32 754	34	28
G12R	GTP	5	32 756	35	28
G12S	GDP	5	32 742	35	28
G12S	GTP	5	32 744	36	28
G12V	GDP	5	32 753	35	28
G12V	GTP	10	32 755	36	28
Wild-type	GDP	5	32 741	35	28
Wild-type	GTP	10	32 740	36	28
Total	(170 µs)	85			

*After energy minimization, the system equilibration was conducted with gradual relaxation in four stages. To obtain individual configurations for production simulations (replicas), an equilibrated system was simulated at 350 K using restraints for K-Ras, waters in the crystal structure, magnesium, and the ligand (GDP or GTP). Every 50 ps one frame was picked as an initial structure for each of the replica simulations.
